# Protein Evolution *via* Amino Acid and Codon Elimination

**DOI:** 10.1371/journal.pone.0010104

**Published:** 2010-04-26

**Authors:** Lise Goltermann, Marie Sofie Yoo Larsen, Rajat Banerjee, Andreas C. Joerger, Michael Ibba, Thomas Bentin

**Affiliations:** 1 Department of Cellular and Molecular Medicine, University of Copenhagen, Copenhagen, Denmark; 2 Department of Microbiology, Ohio State University, Columbus, Ohio, United States of America; 3 Medical Research Council (MRC) Centre for Protein Engineering, Cambridge, United Kingdom; University of Edinburgh, United Kingdom

## Abstract

**Background:**

Global residue-specific amino acid mutagenesis can provide important biological insight and generate proteins with altered properties, but at the risk of protein misfolding. Further, targeted libraries are usually restricted to a handful of amino acids because there is an exponential correlation between the number of residues randomized and the size of the resulting ensemble. Using GFP as the model protein, we present a strategy, termed protein evolution *via* amino acid and codon elimination, through which simplified, native-like polypeptides encoded by a reduced genetic code were obtained *via* screening of reduced-size ensembles.

**Methodology/Principal Findings:**

The strategy involves combining a sequential mutagenesis scheme to reduce library size with structurally stabilizing mutations, chaperone complementation, and reduced temperature of gene expression. In six steps, we eliminated a common buried residue, Phe, from the green fluorescent protein (GFP), while retaining activity. A GFP variant containing 11 Phe residues was used as starting scaffold to generate 10 separate variants in which each Phe was replaced individually (in one construct two adjacent Phe residues were changed simultaneously), while retaining varying levels of activity. Combination of these substitutions to generate a Phe-free variant of GFP abolished fluorescence. Combinatorial re-introduction of five Phe residues, based on the activities of the respective single amino acid replacements, was sufficient to restore GFP activity. Successive rounds of mutagenesis generated active GFP variants containing, three, two, and zero Phe residues. These GFPs all displayed progenitor-like fluorescence spectra, temperature-sensitive folding, a reduced structural stability and, for the least stable variants, a reduced steady state abundance.

**Conclusions/Significance:**

The results provide strategies for the design of novel GFP reporters. The described approach offers a means to enable engineering of active proteins that lack certain amino acids, a key step towards expanding the functional repertoire of uniquely labeled proteins in synthetic biology.

## Introduction

Proteins are usually composed of the 20 naturally occurring amino acids, but variants composed of reduced-size amino acid alphabets have been engineered [1], and the genetic code has been expanded by addition of unnatural amino acids [2], [3]. Global elimination of certain amino acids from a protein can provide important biological insight [4], present altered properties [5], and afford novel options for protein functionalization. Global residue-specific substitutions are possible using unnatural amino acid mutagenesis *via* a codon reassignment strategy [6], albeit with heterogeneous products resulting (*vide infra*). No similar “epigenetic” reassignment strategy exists for substitutions involving canonical amino acids. Designed residue-specific codon elimination may provide a means to produce simplified (encoded and hence homogeneous) variants of natural proteins. But since globular proteins are, in general, only marginally stable [7], such replacements could be at odds with productive protein folding and also hamper function independently. Consistently, the probability that random amino acid replacements will cause protein inactivation averaged ∼34% in the case of human 3-methyladenine DNA glycosylase, and the lac repressor showed similar inactivation frequencies for random mutations [8]. Moreover, targeted mutations in six other proteins showed even higher inactivation frequencies [8]. Finally, experiments on TEM1 β-lactamase and subtilisin suggest that for large numbers of amino acid replacements, there is a negative exponential probability that a protein will retain its structure [9].

Surface-exposed residues may show an increased mutability, as exemplified by the development of “supercharged” green fluorescent protein (GFP) carrying a net charge of +36 or −30 [10] as compared with a net charge of −9 for wild-type GFP [11], [12]. In contrast, substitution of buried (hydrophobic) amino acids is more likely to negatively impact protein folding [13], and such residues therefore evolve at a relatively slow pace [14]. In agreement with these predictions, global residue-specific mutagenesis using codon re-assignment to substitute tri-fluoroleucine for leucine in GFP produced insoluble products. Repeated rounds of directed evolution, however, yielded brightly fluorescent GFP carrying leucine to tri-fluoroleucine substitutions to a level of 77–78% [5]. Based on these findings, we expect global amino acid substitution to significantly impair protein folding and function if this involves replacing multiple buried hydrophobic residues, although there is obviously also a potential for beneficial replacements [15]. To this end, mutations conferring an increased thermodynamic stability enhance a protein's robustness towards random mutations, thereby improving its capacity to evolve (evolvability) as evidenced by studies of cytochrome P450 BM3 mutants [16].

Recent mutagenesis experiments using four different proteins revealed that populations subjected to neutral drifts and purifying selection show increased sequence divergence (including that of buried core residues) in enzymatically active proteins when co-expressed with bacterial GroES/L due to the chaperonin's protein folding buffering capacity [17]. These results confirm and extend earlier observations that chaperonin over-expression masks detrimental genomic mutations in *E. coli* [18]. Similar inferences derive from the capacity of Hsp90 to buffer deleterious mutations in *Drosophila* development [19]. Combining known stabilizing mutations and recombinant chaperone expression could potentially be harnessed to develop a residue-specific global amino acid replacement scheme. In order to explore this approach, which we term protein evolution *via* amino acid and codon elimination, we sought a model protein displaying an easily detectable property and for which such mutations and chaperone activities exist. GFP fulfils these criteria given its autofluorescent properties [12], [20], the existence of so-called superfolder mutations increasing its thermodynamic stability [21], and because non-native GFP is a heterologous substrate of chaperonin GroES/L *in vitro* [22] and in bacteria [23].

## Results and Discussion

**Figure 1:**
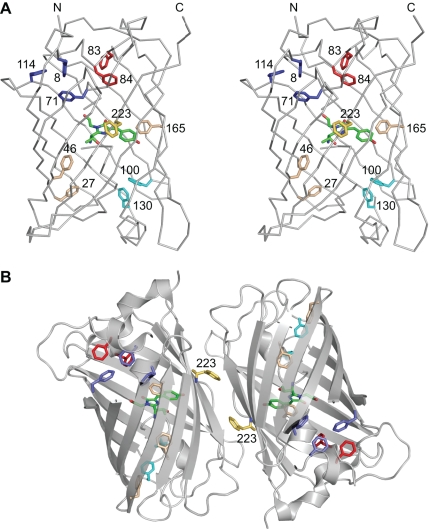
Structure of GFP (PDB entry 2B3Q). (**A**) Stereo view of a GFP monomer. The overall structure of the β-barrel is shown as a Cα trace, with the side chains of Phe residues (11 in total) shown as stick models. The central chromophore moiety is shown in green. Isolated Phe residues inside the β-barrel are shown in beige, the remainder are color-coded according to interactions within local Phe clusters. F223 (shown in yellow) is located at the outside of the β-barrel and is buried within the GFP dimer interface. The benzene ring of F165 is within van der Waals distance to the conjugated ring system of the GFP chromophore. (**B**) Structure of the GFP dimer in the asymmetric unit of PDB entry 2B3Q, shown as semi-transparent ribbon representation. Phe residues and the central chromophore are highlighted as stick models and color-coded as in panel **A**. The figure was prepared using PyMOL (www.pymol.org).

In this work, we used a GFP variant termed GFP-Ref. that closely resembles the previously described folding reporter GFP [24] as a starting point for mutagenesis (Methods S1). GFP-Ref. contains a total of 11 Phe residues that are spread throughout the 238 amino acid β-barrel structure of the protein, at varying distance from the central chromophore. One Phe residue (F223) is located at the surface, whereas the remaining 10 phenylalanines are buried within the hydrophobic core of the β-barrel (Fig. 1), which is reflected in their very limited solvent accessibility (Fig. S1).

**Figure 2:**
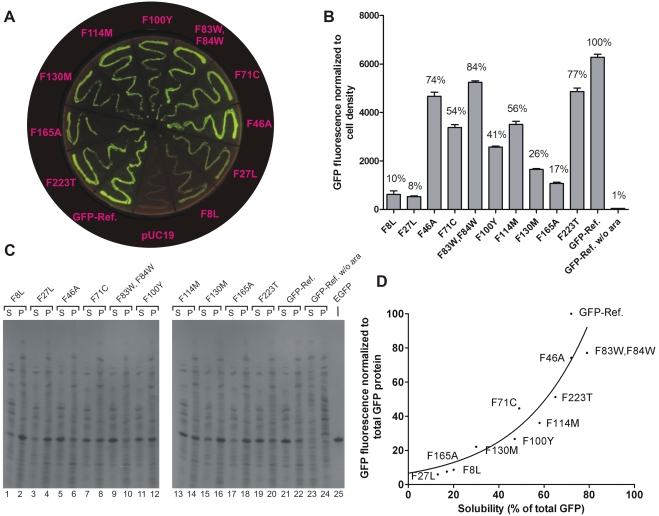
Single-substitution GFP mutants. (**A**) Fluorescence image showing streaks of the indicated constructs transformed into DH5α and grown at 37°C. (**B**) Quantification of fluorescence from DH5α cultures expressing the indicated GFPs. Fluorescence and cell growth was monitored over time (8 h) at 37°C in the presence of inducer (0.1% arabinose, ara), and the end level fluorescence was normalized against cell density. Background fluorescence provided by a pUC19/DH5α culture was subtracted. Cell growth occurred at similar rates for the different mutants (Fig. S2). The mean and standard deviation (SD) of triplicate experiments is shown. (**C**) SDS-PAGE analysis of cell-free extracts. S (soluble fraction), P (insoluble fraction). GFP was located using commercial EGFP as a marker (lane 25). (**D**) Fluorescence versus solubility for the indicated constructs. Data points were fitted to an exponential fit using Prizm software v. 5.0.

Simultaneous randomization of all 11 GFP-Ref. Phe residues with the 19 non-Phe amino acids would involve an ensemble of 19^11^ (1×10^14^) protein variants (56^11^ at the DNA level with our codon usage, *vide infra*), a number too large to screen exhaustively by current methodology. Instead, we first probed the importance of individual Phe residues for GFP-Ref. fluorescence using saturation mutagenesis and then combined the best performing amino acid replacements into a globally modified construct. A set of parallel reactions was set up, each substituting single UUU or UUC (Phe) codons with NBR and NVN libraries (where N = A, C, G, or T; B = C, G, or T; R = A or G; V = A, C, or G), hence encoding all canonical amino acids except phenylalanine. This approach yields 19 variants per amino acid position under query, and 19^2^ for positions F83 and F84, which were mutagenized simultaneously. All Phe residues could be individually replaced (Fig. 2A), but with substantial and variable fluorescence reductions ensuing (fluorescence ranged from 8% to 84% of parental GFP-Ref.) (Fig. 2B). Each position displayed different preferences with respect to the physico-chemical properties of amino acid substitutions including size, polarity and aromaticity (Table S1, and Fig. S2). Not surprisingly, neither acidic (D or E), basic (K, R or H), or large polar amino acids (N or Q) emerged from the screen for Phe replacements. To address possible causes of the fluorescence fluctuations observed with these single-substitution GFP mutants, whole cell lysates were analyzed by SDS-PAGE and Coomassie staining. Differences in GFP abundance were minor and did not correlate well with fluorescence variations (Fig. S3A). In contrast, GFP solubility correlated strongly with fluorescence (Fig. 2C–D), indicating that phenylalanine, like leucine [5], plays important roles in GFP folding.

### Evolution of Phe-free GFP

**Figure 3:**
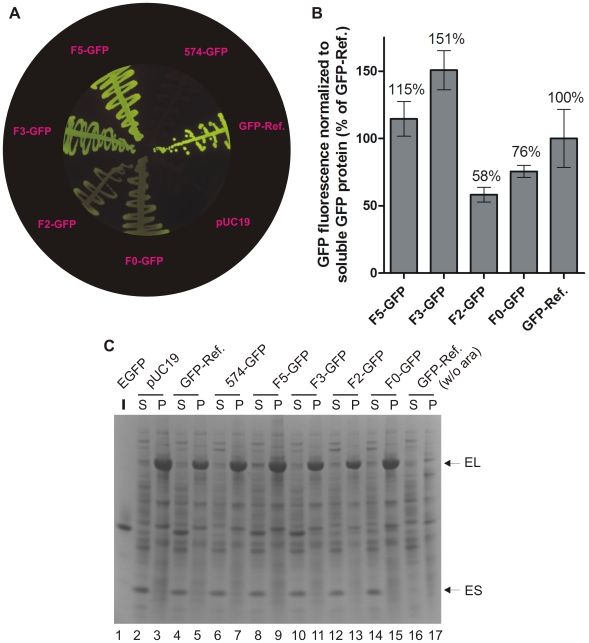
Evolution of GFP devoid of phenylalanines. (**A**) Streaks of the indicated GFP mutants induced at room temperature and co-expressed with GroEL/S. (**B**) Quantification of fluorescence from DH5α cultures expressing the indicated GFPs. After induction, fluorescence and cell growth was monitored over time (18 h) at 23°C in the presence of inducer (ara), and the end level fluorescence was normalized against soluble GFP protein and corrected for background fluorescence as in Fig. 2. The mean and SD of quadruplicate experiments is shown. (**C**) SDS-PAGE analysis of cell-free extracts as described in the legend to Fig. 2.

Combination of the most active single-substitution GFP variants yielded 574-GFP (Table 1), which was entirely devoid of phenylalanine residues but also resulted in non-fluorescent colonies. Restoration of fluorescence required combinatorial re-introduction of phenylalanine residues at five positions (F8, F27, F71, F130 and F165) yielding F5-GFP (Table 1, see Fig. 3 and Fig. S4 for fluorescence and expression data of 574-GFP and F5-GFP under different conditions and *vide infra*). To enable development of GFP variants carrying further Phe substitutions, the temperature of gene expression was reduced, a common approach in recombinant protein production [25]. This afforded fluorescent F3-GFP carrying F27, F130 and F165 (Table 1). Attempts to produce functional GFP by combinatorial mutagenesis of these remaining Phe residues using reduced temperature of gene expression proved futile. We speculated that the folding capacity of the GFP mutants with reduced Phe content could be enhanced by chaperone complementation. To investigate if the evolved GFP variants were substrates of GroES/L, similar to wild-type GFP from *Aequorea victoria* [23], the DH5α strains expressing GFP-Ref., 574-GFP, F5-GFP and F3-GFP were supplemented with pGro7, a compatible plasmid expressing chaperonin GroES/L from P_bad_ or a control plasmid pACYC184, and expressed at two different temperatures (Fig. S4A and B). Remarkably, while streaks of bacteria expressing F3-GFP showed only minor fluorescence differences with and without chaperonin co-expression at room temperature, GroES/L complementation rescued a pronounced temperature sensitivity at 37°C. In contrast, F5-GFP showed only marginal chaperonin complementation at 37°C and none at room temperature. Neither GFP-Ref. nor 574-GFP were visibly influenced by GroES/L at either temperature (Fig. S4A and B). These data argue that GFP-Ref. is either independent or only weakly GroES/L dependent, consistent with the folding-optimized properties at 37°C of its precursor, “cycle 3” GFP [26] (the relation of these GFPs is described in the Methods S1). Furthermore, 574-GFP fluorescence could not be revived under any conditions tested (Fig. 3A, Fig. S4A and B), and its expression produced comparably low levels of protein (Fig. 3C), suggesting increased sensitivity towards proteolytic degradation as a result of non-productive GFP folding. Continued screening at reduced temperature and with GroES/L complementation yielded fluorescent F2-GFP carrying F27 and F165 (Fig. 3A and B, see Table 1). Even with chaperonin co-expression, fluorescence could only be achieved when expressed at room temperature (Fig. S4A and B). Finally, introduction of five previously described superfolder mutations (S30R, Y39N, N105T, I171V, and A206V) [21] into the F2-GFP scaffold enabled evolution of fluorescent GFP variants with zero Phe residues (F0-GFP; Fig. 3A and B, see Table 1). Quantitative analysis of cell-free extracts from cultures expressing F5-GFP through F0-GFP showed considerable differences in protein abundance (Fig. S3B) and solubility (*vide infra*). Consequently, fluorescence data were normalized to the amount of soluble (*i.e.* folded) GFP protein (Fig. 3C). The fluorescence levels of F2- and F0-GFP were 58% and 76% of GFP-Ref. when normalized to protein abundance (Fig. 3B), respectively, indicating that the chromophore environment had been only marginally perturbed by global Phe elimination. Most GroEL appeared to be insoluble, whereas most GroES was soluble in all of the present conditions (Fig. 3C). This contrasts with previous work in which most recombinant GroEL was soluble using pGro7 in combination with pET32(b) derivatives in *E.coli* BL21(DE3) [17]. Our result is reproducibly seen in three different strain backgrounds, and with different levels of inducer (data not shown), so currently we have no explanation for this discrepancy. In any case, this suggests that considerable optimization is still possible. Finally, F0-GFP, when co-expressed with GroES/L, produced fluorescent cultures in two additional bacterial strain backgrounds (DH10B and BL21(DE3)), showing that F0-GFP maturation was not linked to a particular genotype (Fig. S5).

**Table 1:** Phenylalanine substitutions in the evolved GFP variants.

Position	% ASA	Single-substitution aa	574-GFP	F5-GFP	F3-GFP	F2-GFP	F0-GFP*	Phylogen. variation	Phylogen. consensus
F8	2	L,M,Y	L	F	L	L	L	I,L,V	I
F27	2	L	L	F	F	F	W	F	F
F46	1	A,V,T,I,G	A	A	A	A	A	L,I,V	L
F71	0	C,L,M,V,A	C	F	L	L	L	F,Y	F
F83,F84	0,0	W,W;W,L;W,M	W,W	W,W	W,W	W,W	W,W	Y,F,I; F,L,V,K	Y,F
F100	2	Y,W	Y	Y	Y	Y	Y	F,Y	F
F114	16	M,L,W,I,Y,V,K	M	M	M	M	M	L,M,V,I,F	L
F130	5	L,M,I	M	F	F	V	L	F,L	F
F165	9	A,M,W,Y,L,T	A	F	F	F	I	V,I,S,D,N,L,R,C,F	V
F223	5	T,V,M,S,A,G	T	T	T	T	T	H,T,V,K,N,D,I,Y,S,A,F	H

(*) F0-GFP derives from one of nine independent colonies examined (containing plasmids p607-c1 through c9) all devoid of phenylalanine and fluorescent to different extents. In addition to the F27W/F165I F0-GFP variant investigated, four alternative fluorescent F0-GFP sequences were found with the mutations F27W/F165C; F27I/F165Y; F27V/F165W and F27W/F165V. Phylogenetic (Phylogen.), Amino acid (aa).

### GFP retains structure and function when encoded by 19 amino acids

**Figure 4:**
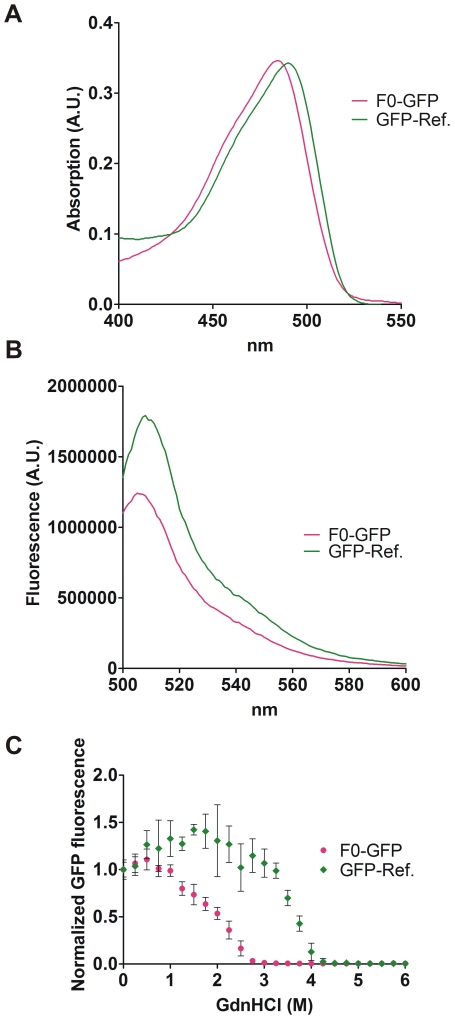
Biophysical characterization of evolved F0-GFP. (**A**) Absorption and (**B**) emission spectra for F0-GFP versus GFP-Ref. (**C**) GdnHCl-induced protein unfolding at 72 h of incubation. The mean and SD of triplicate experiments is shown.

Biophysical characterization of Ni-NTA agarose purified GFP variants revealed that the absorption maximum was shifted to 485 nm for F0-GFP similar to superfolder GFP [21], as compared to 490 nm for GFP-Ref. (Fig. 4A). All mutants investigated displayed fluorescence emission spectra with a maximum emission at 508 nm when excited at 480 nm, similar to GFP-Ref (Fig. 4B and Fig. S6A).

Protein stability was investigated by guanidine hydrochloride (GdnHCl) unfolding titrations (Fig. 4C and Fig. S6B and C). GFP is known to show non-equilibrium behavior in denaturant-induced unfolding [27] (consistent with the unfolding transitions shifting towards lower Gdn-HCl concentrations at increased incubation time (cf. Fig. S6B and C)), so true free energies of unfolding cannot be deduced from unfolding transitions alone. However, such unfolding transitions provide [GdnHCl]_50_ values (*i.e.* the denaturant concentration yielding 50% unfolding under a given set of conditions), allowing direct comparison of the kinetic stability against unfolding of our GFP mutants. [GdnHCl]_50_ values were ∼3.7 M and ∼2.2 M for GFP-Ref. and F0-GFP, respectively, clearly demonstrating a destabilization of the GFP variant devoid of Phe residues (Fig. 4C). The remaining GFP mutants with reduced Phe-content (F5-GFP, F3-GFP and F2-GFP) also showed increased sensitivity towards denaturant (Fig. S6B and C). For some of the investigated GFP mutants, addition of limited amounts of denaturant resulted in an increase of fluorescence (as also reported for EGFP [28]), and this was particularly noticeable for the F3-GFP 72 h samples (Fig. S6C). Such increases could result from an altered chromophore environment, but elucidation of the detailed molecular background for this observation requires further experiments. It is also interesting to note that the large stability difference between F3-GFP and F2-GFP (Fig. S6) is caused by a single-substitution (F130V in the tested variant, F130L and F130I in the other two selected variants). A similar stability loss upon substitution of a buried phenylalanine by a smaller hydrophobic residue has, for example, been observed for an oncogenic, cavity-creating mutation (F270L) in the tumor suppressor p53 protein [29].

### Protein evolution *via* amino acid and codon elimination

Here we were able to harness thermodynamic stabilization [16] and chaperonin over-expression [17] to evolve novel native-like proteins, in this case GFP variants, with progressively diminished Phe content. Given the impact of each single Phe mutation on protein folding and fluorescence, it is somewhat surprising that a viable variant entirely devoid of Phe residues could be evolved. The thermodynamic stability of F0-GFP could be optimized by introduction of compensatory changes, either through structural considerations or *via* directed evolution to reduce or eliminate the chaperonin dependency and fluorescence temperature sensitivity. Additional rounds of randomization could, for example, target clustered phenylalanine positions in combination (e.g. residues 8, 71 and 114) (Fig. 1) and also include residues in the immediate environment of the original Phe positions to improve packing interactions and hence protein stability (taking into account that libraries expand exponentially with the number of amino acid positions simultaneously targeted).

Phylogenetic analysis of 27 members of the GFP family in the Sanger Institute Pfam database (entry PF01353) revealed variable conservation of Phe residues (Table 1, columns 9 and 10). Comparison of phylogenetic variation with sequences obtained by experiment (Table 1) shows that several amino acid substitutions in F0-GFP, including F27W, F46A, and the F83W/F84W combination, could not have been anticipated based on sequence alignments alone, similar to what was observed for the 57 residue β-barrel-like src SH3 domain [30], hence supporting a fully random mutagenesis approach. Nevertheless, phylogenetic analysis would have enabled identification of viable substitutions for several Phe positions targeted in the different GFP constructs made, indicating its utility in combination with random approaches. The only non-hydrophobic Phe substitution in F0-GFP, F223T, was found for a residue that is not located in the hydrophobic core of the β-barrel but on its surface, forming part of the dimer interface observed in some crystal structures (Fig. 1B). Incidentally, this residue exhibits the largest phylogenetic variability among the residues that were targeted for randomization (Table 1). It is also interesting to note that substitution of the only strictly conserved Phe residue, F27, resulted in the lowest fluorescence activity observed among all single-substitution variants (Fig. 2). Not surprisingly, it was one of two residues, together with F165, requiring addition of superfolder mutations [21] to generate F0-GFP, highlighting the structural preference for a phenylalanine at this position. Contrary to F27, F165 shows a large phylogenetic variability, yet its replacement required additional fine-tuning, probably because of its location in direct vicinity of the conjugated chromophore ring system (Fig. 1).

We have found several active GFP variants devoid of phenylalanine (see legend to Table 1) using a reductive approach and by screening of a very limited number of colonies (<100.000 cfu). Clearly, there is a potential for finding superior Phe-lacking GFP variants by screening of a fully Phe mutagenized library but such an ensemble is well beyond the current capacity of bacterial systems, which we estimate to ≤10^9^. Introduction of super folder mutations [21] into the starting construct and use of chaperonin complementation [17] from the very beginning could help “bridge the gap”, enabling selection of active Phe-lacking GFP variants from a reduced-size tractable ensemble. Importantly, such experiments might also allow quantification of the extent to which stabilizing mutations, chaperonin complementation, temperature etc., modify protein neutral networks (those sequences that map to a GFP structure that provides green fluorescence) and hence contribute to an integrated description of how such parameters impact protein evolution. Finally, structure-based methods for sequence engineering [31], [32] may be implemented into this approach to identify replaceable positions and to limit libraries to manageable ensembles, which would allow further optimization of protein stability. Our screening and selection system could also be adjusted to select for GFP variants with altered spectral properties.

The *in vivo* data suggest that a major component of the fluorescence temperature sensitivity seen on agar plates is mechanistically linked to a (kinetic) folding problem since fluorescent colonies remained fluorescent even after transfer from permissive to non-permissive temperature and continued incubation (Fig. S4C). While the steady state GFP protein levels were close to constant for the single-substitution GFP mutants and reduced fluorescence correlated with increased protein aggregation (Fig. 2), the more heavily substituted F2-GFP and F0-GFP variants in particular showed a substantially reduced abundance (Fig. 3 and Fig. S3). Because GFP expression is regulated by the same promoter in all constructs, and because the regions most important to bacterial translation (including the ribosome binding site and its immediate vicinity [33] as well as the 5′ coding region [34]) was preserved during mutagenesis, the latter observations are most simply explained by an increased degradation of these species. Hence, these GFP mutants display both impaired folding- and/or reduced biological stability.

Several studies have focused on reducing the amino acid alphabet, and in an extreme case a functional chorismate mutase composed of only 9 different amino acids [35] was achieved using binary patterning [36] and iterated screens. The present data suggest that such endeavors could be greatly facilitated by the implementation of chaperonin complementation and stabilizing mutations in order to expand the neutral networks of protein folding.

We envision that our GFPs might find applications as sensors of translational misreading [37] and as folding reporters [24], with the added benefit that translation errors (in this case those involving tRNA^Phe^) can now be limited to pre-defined positions through codon elimination. Furthermore, our findings are potentially significant for future synthetic biology applications because they open up the possibility for re-engineering of variants of natural proteins into which unique chemical functionalities can then be introduced, for instance using codon reassignment and unnatural amino acid mutagenesis [6], [2]. More broadly, application of the principles described here can now be extended to other proteins with substantially increased capacities for structural and functional re-engineering.

## Materials and Methods

### Plasmid construction

For construction of single-substitution GFP mutants, each phenylalanine position in GFP-Ref. (encoded by p369-c1, GenBank accession number GU994007) was mutagenized by “divergent PCR” using p369-c1 (Methods S1) as a template and one of two forward primers containing 5′-NBR or 5′-NVN extensions and a juxtaposed reverse primer (Table S2). PCR was performed using Accupol DNA polymerase (Ampliqon). The PCR product was treated with *Dpn*I and subjected to a second round of PCR using primers 5′ phosphorylated using polynucleotide kinase (Fermentas) and ATP. The PCR product was circularized using T4 DNA ligase (Fermentas) and transformed into chemically competent *E.coli* DH5α cells. Fluorescent colonies were selected from LB-agar plates containing 100 µg/ml ampicillin and 0.2% arabinose by visual screening using a Dark Reader (Clare Chemicals). A complete screen of the single-substitution GFP mutants was carried out (except for F83/F84 where only 202 cfu were required to find the most active mutant among all the single-substitution variants). 574-GFP (encoded by p574-c20, GU994008) was constructed by gene assembly using oligonucleotides of ∼50 nt overlapping by 20–30 bases, and external 5′-biotinylated primers otb141 and otb151 for amplification (Table S2 and Table S3). The PCR product was purified using S300 size exclusion spin columns (GE Healthcare), *Nde*I-*EcoR*I restriction digested, purified using streptavidin magnetic particles (Roche) and ethanol precipitation, and cloned into identically digested p338-c17 (see Methods S1). F5-GFP (encoded by p582-c30, GU994009) was constructed using the oligonucleotides listed in Table S2 and the Multi Quick Change Mutagenesis Kit (Stratagene). Codons encoding Phe were re-introduced at 3–5 positions in different combinations resulting in a total of 218 colonies. Only a single fluorescent colony was identified on a plate containing 33 colonies and deriving from a mutagenesis reaction targeting 5 residues. Libraries for F3-GFP (encoded by p610, GU994010) and F0-GFP (encoded by p607-c3, GU994012) were constructed by gene assembly (see Table S2 and Table S3) as described for p574-GFP and using p574-c20 (producing a non-fluorescent background in the presence of inducer) for vector preparation. For identification of F3-GFP, ∼6×10^4^ colonies were screened. F2-GFP (encoded by p611, GU994011) was constructed by “divergent PCR” as described above using p610 as a template and oligonucleotides listed in Table S2 and identified from a screen of 316 colonies. Three libraries were constructed for F0-GFP using different F2-GFP variants (F130L, I or V) (Table S2 and S3). Fluorescent F0-GFPs (see legend to Table 1) as identified by screening of >3000 colonies, all derived from the F130L variant. GroES/L complementation was provided by co-transformation of the pACYC184 based pGro7 plasmid (named p544 in our inventory) from Takara Biosciences. Transformants were grown overnight at 37°C on nitrocellulose filters on LB-agar plates with 100 µg/ml ampicillin and 40 µg/ml chloramphenicol. Filters were transferred to plates containing antibiotics and 0.1% arabinose for induction and incubated at room temperature. Histidine affinity tagged vectors were constructed by PCR amplification of inserts from p369-c1, p582-c30, p610, p611 and p607-c3 using otb141 and otb558 and inserted into the *Nde*I-*Eco*RI sites of p581-c31 as described above, hence generating p612-c3, p614-c2, p615-c2, p616-c3, and p617-c3 expressing His6-tagged variants of GFP-Ref., F5-GFP, F3-GFP, F2-GFP, F0-GFP, respectively. Constructs were purified by minipreparation using the GeneJet kit (Fermentas) and sequenced using primer otb164 and the sequencing service at Macrogen Korea.

### Fluorescence Measurements

Starter cultures of cells containing single-substitution GFP constructs were inoculated from frozen glycerol stocks into 96-well microtiter plates containing 200 µl/well LB-broth supplemented with 100 µg/ml ampicillin. After O.N. incubation at 37°C with shaking (high linear mode in a TECAN GENios microtiter plate reader), the starter cultures were re-inoculated at 100-fold dilution into LB-broth containing 100 µg/ml ampicillin and 0.1% arabinose. Measurements were carried out on living cells at 37°C every 20 min for a period of up to 18 hours with intermediate shake cycles in linear mode. Cell cultures were allowed a lag phase of 200 s after each shake cycle before measurement. Optical density was measured at 595 nm. GFP was excited at 480 nm and fluorescence was recorded at 520 nm using an integration time of 20 µs. In the case of F5-GFP through F0-GFP co-expressing GroES/L, cultures were grown at 37°C until reaching an OD of 0.5–0.7 and then induced by addition of arabinose to a final concentration of 0.1%. Subsequent fluorescence and absorbance measurements were done for 18 h at 23°C

### Assessment of protein solubility in *E. coli*

Cell-free extracts for solubility analysis were prepared by harvesting an amount of overnight culture corresponding to OD_595_ = 1.8 in 100 µl at 20,000 g for 15 min (no leaking of fluorescence into the medium was detected). The soluble protein fraction was obtained by incubating resuspended cell pellets in 40 µl B-PER (PIERCE) containing 10 µg/ml DNase I for 10 min. at room temperature followed by centrifugation at 20,000 g for 12 min. The supernatant was transferred to a fresh tube and the pellet re-extracted as above followed by pooling of supernatant fractions. The final pellet containing the insoluble protein fraction was re-suspended in 80 µl B-PER supplemented with DNaseI as above. All fractions were supplemented with 20 µl 5 x SDS-loading buffer and heated to 90°C for 2 min. and subsequently analyzed using NuPAGE 4–12% Bis-Tris gels (Invitrogen) followed by staining with PageBlue (Fermentas). Gels were analyzed using TotalLab TL100 or ImageQuant version 5.1 software.

### Protein absorbance measurements

The absorbance of purified protein samples was measured from 200–600 nm using a Shimadzu UV-1700 UV-Vis spectrophotometer with 1 cm path length. Extinction coefficients at 280 nm for GFP-Ref. (22000 M^−1^ cm^−1^) and F0-GFP (31543 M^−1^ cm^−1^) were calculated using the ProtParam application on the ExPASy proteomics server.

### Emission spectra

Affinity purified GFP-Ref., F5-GFP, and F3-GFP were diluted to obtain an OD_488_ identical to that of F0-GFP. The samples were then diluted ∼660-fold in dialysis buffer for fluorescence measurements (excitation 480 nm, emission 510 nm). F2-GFP was obtained at reduced yield and therefore diluted only ∼55-fold. Fluorescence was measured using a Fluorolog-3 spectrofluorimeter (Horiba Jobin Yvon), with a 3 mm path length cuvette to avoid inner filter effects, and using 5 nm slit width for excitation and emission, and a 1 nm step size.

### Unfolding

GFP variants were incubated at room temperature with increasing concentrations of guanidine hydrochloride (GdnHCl) from 0–6 M in unfolding buffer (40 mM Tris-HCl pH 7.5, 200 mM NaCl). Emission spectra were measured after 24 h and 72 h. The fraction of unfolded protein was calculated by integration of the emission spectra from 500 nm to 650 nm as compared to samples without GdnHCl. Protein concentrations for unfolding titrations were ∼0.0025 mg/ml as calculated based on ε_280_. All measurements were carried out at least three times.

### Calculation of solvent accessibility

Solvent accessibility of GFP residues was calculated using the program ASA-view [38].

### Phylogenetic variation

Phylogenetic variation and phylogenetic consensus sequences (Table 1) were determined by analysis of 27 members of the GFP family in the Sanger Institute Pfam database entry PF01353 using Jalview software from the Janelia farm research campus at http://pfam.janelia.org//family/PF01353 [39].

## Supporting Information

Methods S1 Supporting methods for protein evolution via amino acid and codon elimination.
(0.05 MB DOC)

Table S1 Amino acid substitutions and in vivo GFP fluorescence for all identified single-substitution GFP mutants. a) Nomenclature: individual constructs are identified by a double digit number (where the first digit indicates whether NBR (#1) or NVN (#2) primers were used, and the second digit indicates numerically the phenylalanine residue counting from the N-terminus of GFP) followed by a dash and a colony number, i.e., 21–115 represents colony 115, which originated from a screen using a NVN-library primer at the first phenylalanine residue F8. b) GFP fluorescence end level normalized to cell density (duplicate experiments). c) Standard deviation. The data were corrected for background fluorescence using a pUC19/DH5α culture. *) Asterisk indicates the single-substitution GFP mutants compiled in Figure 2. Data from Figure S2 was used.
(0.01 MB PDF)

Table S2 Oligonucleotides used in this study.
(0.17 MB DOC)

Table S3 Oligonucleotide combinations for construction of GFPs with reduced Phe content by gene assembly. The numbers indicated for forward (column 1) and reverse (column 2) oligonucleotides are defined in Table S2. “Phe-residue” in column 3 indicates which Phe-codon(s) in GFP-Ref. that is covered by the oligonucleotide in question. The (−;−) notation signifies forward (left dash) and reverse (right dash) oligonucleotide. The column entitled “substitution” states whether the given oligonucleotide contains the original Phe-codon or a substitution. See Materials and Methods for details.
(0.41 MB DOC)

Figure S1 Amino acid solvent accessibility in GFP. Solvent accessibility analysis of amino acids in folding reporter GFP (PDB file 2B3Q) using ASAview software. The global count of each amino acid is given below the x-axis. Amino acid colour code: hydrophobic (grey), cystein (yellow), polar uncharged (green), positive (blue), and negative (red).
(0.74 MB TIF)

Figure S2 In vivo GFP fluorescence accumulation and growth curves for all single-substitution mutants analyzed. Overnight starter cultures were diluted 100-fold, into LB-amp supplemented with 0.1% arabinose and grown for 8 h at 37° C. All measurements were performed in duplicates and the mean and SD for each data point is shown.
(0.19 MB PDF)

Figure S3 GFP abundance in whole cell lysates. Protein analysis by SDS-PAGE and coomasie staining of whole cell lysates from cultures expressing (A) single-substitution GFP mutants and (B) evolved GFP variants. EL and ES indicates GroEL and GroES, respectively.
(3.01 MB TIF)

Figure S4 Chaperonin and temperature dependence of evolved GFP variants. DH5α expressing the indicated evolved GFPs and co-transformed with either pGro7 or pACYC184 were streaked on nitrocellulose placed on LB-agar plates containing ampicillin and chloramphenicol and grown overnight at 37° C. The filters were transferred to similar plates supplemented with 0.1% arabinose and incubated overnight at 37° C (A) or room temperature (B). (C) GFP mutants expressed at room temperature in the presence of GroES/L followed by transfer to 37° C and continued incubation for 24 h. pUC19 was used as a control as indicated.
(5.64 MB TIF)

Figure S5 F0-GFP and GFP-Ref expression in three different strains. Fluorescence of overnight cultures co-expressing the indicated GFP variant and GroES/L in *E.coli* strains DH5α, BL21(DE3) and DH10B. Fluorescence and cell growth was monitored over time (18 h) at 23° C in the presence of 0.1% arabinose and the end level fluorescence was normalized against soluble GFP protein. Background fluorescence using a pUC19/DH5α culture was subtracted. The mean and SD of quadruplicate experiments is shown.
(1.63 MB TIF)

Figure S6 Biophysics of GFP Phe mutants. (A) Emission spectra of indicated GFP variants. (B and C) GdnHCl-unfolding titration at room temperature of the indicated GFP variants at 24 h (B) or 72 h (C) of incubation. The mean and SD of triplicate experiments is shown.
(2.41 MB TIF)
