# Supplementary material for: Protein Evolution via Amino Acid and Codon Elimination
Source: PLoS One. 2010 Apr 26;5(4):e10104. doi: 10.1371/journal.pone.0010104 (PMC2859931; doi:10.1371/journal.pone.0010104)
Supplement: Methods S1 — Supporting methods for protein evolution via amino acid and codon elimination. (0.05 MB DOC) [file pone.0010104.s001.doc]

**Methods S1.**

**Protein Evolution *via* Amino Acid and Codon Elimination**

Lise Goltermann^1^, Marie Sofie Yoo Larsen^1,4^, Rajat Banerjee^2^, Andreas C. Joerger^3^, Michael Ibba^2^, and Thomas Bentin^1^*

^1^ Department of Cellular and Molecular Medicine, University of Copenhagen, Copenhagen, Denmark.

^2^ Department of Microbiology, Ohio State University, Columbus, Ohio, USA

^3^ MRC Centre for Protein Engineering, Cambridge, United Kingdom

^4^Current address: Biotech Research Innovation Centre, University of Copenhagen, Copenhagen, Denmark

*) Corresponding author

**Construction of p369-c1**. Wild type GFP contains 13 Phe residues [1]. Folding reporter (FR)-GFP [2] harbouring the “cycle 3” (F99S, M153T, V163A) [3], F64L/S65T [4], and the silent Q80R [5] mutation contain 11 phenylalanines. The GFP providing a starting point for all mutagenesis in this paper is termed GFP-Ref. GFP-Ref. is closely related to FR-GFP but does, however, not contain the synthetic 12 amino acid N-terminal extension of FR-GFP. Furthermore, GFP-Ref./FR-GFP use synonymous codons at position Lys41 (AAG/AAA), Leu42 (CTT/CTC), Thr65 (ACT/ACC).

The p369-c1 plasmid (we use serial numbers for construct identification followed by a colony number for distinction among colony forming units, if available) carries a pBR322 origin of replication, a *bla* gene for ampicillin selection, and expresses GFP-Ref. directed by the Pbad promoter/AraC regulator system [6]. p369-c1 was generated using the previously described pBAD-GFPuv plasmid (named p335 in our inventory) expressing “cycle 3” GFP as a template (Genbank accession no: CVU62637). pBAD-GFPuv restriction sites *Nde*I (map positions 1575 and 4928), and *Bam*HI (map positions 1865, and 2085) were first rendered non-functional using oligonucleotides otb134-otb137 (Table S2) and the QuickChange Multi Site-Directed Mutagenesis Kit (Stratagene) resulting in plasmid p338-c17 (synonymous codon usage was employed for restriction sites located inside GFP). p338-c17 was identified by its ability to provide ampicillin resistance to transformed bacteria, by visual identification of UVA excitable green fluorescent colonies on LB-agar supplemented with arabinose, and by restriction mapping of purified plasmid. The GFP F64L, S65T mutations (4) were subsequently introduced using oligonucleotide otb177. For historical reasons, these latter mutations were first produced in a different (smaller) plasmid, p365-c1. Subsequently, the entire GFP gene was excised from p365-c1 using *Nde*I and *Eco*RI and ligated into corresponding restriction sites in p338-c17 hence generating p369-c1. p369-c1 was sequenced using otb164 (Table S2) as a primer.

**Protein expression and purification**. Single colonies co-transformed with plasmids expressing affinity tagged versions of GFP-Ref., or F5-GFP through F0-GFP and pGro7 encoding GroES/L, were inoculated and grown overnight without induction as starter cultures in **~**5 ml LB containing 100 µg/ml ampicillin and 25 µg/ml chloramphenicol at 37°C. The starter cultures were used to inoculate 1 liter cultures, which were incubated at 37°C for 2½ h before addition of 5 ml 20% arabinose to a final concentration of 0.1% and incubation continued at room temperature overnight. All subsequent handling was performed on ice or at 4°C. Cells were harvested by centrifugation at ~5000 g for 6 min and lyzed by sonication in 30 ml sonication buffer containing protease inhibitor (1 protease inhibitor tablet per 80 ml sonication buffer: 50 mM Tris-HCl pH 8, 10 mM imidazol, 1 mM β-mercaptoethanol, 100 mM NaCl, 10% glycerol). Soluble protein was collected by centrifugation at ~44,000 g for 50 min. The supernatant was purified on Ni-NTA agarose. Fractions containing GFP as determined by SDS-PAGE were pooled and dialyzed twice against 1 liter dialysis buffer (20 mM Tris-HCl pH 8, 1 mM EDTA, 1 mM β-mercaptoethanol, 100 mM NaCl, 10% glycerol). Samples were concentrated to ~1/3 volume in dialysis buffer supplemented with glycerol to a final concentration (v/v) of 50%.

**Calculation of solvent accessibility**. Solvent accessibility of GFP amino acid residues (incl. side chain and backbone surface areas) was calculated using the program ASA-view [7] available at (<http://www.netasa.org/asaview/>). As the default, ASAview reports relative accessible surface areas normalized to a value of 1.

References

1. Prasher DC, Eckenrode VK, Ward WW, Prendergast FG, Cormier MJ (1992) Primary structure of the Aequorea victoria green-fluorescent protein. Gene 111: 229-233.

2. Waldo GS, Standish BM, Berendzen J, Terwilliger TC (1999) Rapid protein-folding assay using green fluorescent protein. Nat Biotechnol 17: 691-695.

3. Crameri A, Whitehorn EA, Tate E, Stemmer WP (1996) Improved green fluorescent protein by molecular evolution using DNA shuffling. Nat Biotechnol 14: 315-319.

4. Cormack BP, Valdivia RH, Falkow S (1996) FACS-optimized mutants of the green fluorescent protein (GFP). Gene 173: 33-38.

5. Chalfie M, Tu Y, Euskirchen G, Ward WW, Prasher DC (1994) Green fluorescent protein as a marker for gene expression. Science 263: 802-805.

6. Guzman LM, Belin D, Carson MJ, Beckwith J (1995) Tight regulation, modulation, and high-level expression by vectors containing the arabinose PBAD promoter. J Bacteriol 177: 4121-4130.

7. Ahmad S, Gromiha M, Fawareh H, Sarai A (2004) ASAView: database and tool for solvent accessibility representation in proteins. BMC Bioinformatics 5: 51-
