# Supplementary material for: Protein Evolution via Amino Acid and Codon Elimination
Source: PLoS One. 2010 Apr 26;5(4):e10104. doi: 10.1371/journal.pone.0010104 (PMC2859931; doi:10.1371/journal.pone.0010104)
Supplement: Table S1 — Amino acid substitutions and in vivo GFP fluorescence for all identified single-substitution GFP mutants. a) Nomenclature: individual constructs are identified by a double digit number (where the first digit indicates whether NBR (#1) or NVN (#2) primers were used, and the second digit indicates numerically the phenylalanine residue counting from the N-terminus of GFP) followed by a dash and a colony number, i.e., 21–115 represents colony 115, which originated from a screen using a NVN-library primer at the first phenylalanine residue F8. b) GFP fluorescence end level normalized to cell density (duplicate experiments). c) Standard deviation. The data were corrected for background fluorescence using a pUC19/DH5α culture. *) Asterisk indicates the single-substitution GFP mutants compiled in Figure 2. Data from Figure S2 was used. (0.01 MB PDF) [file pone.0010104.s002.pdf]

clone number[a]	aa substitution	codon substitution	GFP fluo.[b]	SD[c]	%GFP-Ref.
GFP-Ref.			6280	125	100
uninduced GFP-Ref.			45	56	1
11-25*	F8L	UUC->CTA	621	150	10
11-37	F8M	UUC->ATG	547	30	9
11-38	F8L	UUC->TTG	313	29	5
11-40	F8L	UUC->CTG	485	28	8
21-115	F8Y	UUC->TAC	140	24	2
12-41*	F27L	UUU->CTG	532	28	8
13-1	F46T	UUU->ACA	984	34	16
13-2	F46V	UUU->GTA	1689	94	27
13-3	F46T	UUU->ACG	1300	42	21
13-4	F46I	UUU->ATA	1277	36	20
23-91*	F46A	UUU->GCG	4672	170	74
23-104	F46G	UUU->GGT	252	25	4
14-5	F71V	UUU->GTG	523	31	8
14-6	F71L	UUU->TTA	1918	45	31
14-7	F71L	UUU->CTA	1773	43	28
14-8	F71V	UUU->GTA	1130	42	18
24-113	F71A	UUU->GCC	36	17	1
24-117	F71L	UUU->TTA	2069	106	33
24-118	F71C	UUU->TGT	3254	155	52
24-119	F71M	UUU->ATG	1939	86	31
24-120*	F71C	UUU->TGT	3381	120	54
24-105	F71C	UUU->TGT	3278	117	52
15-66	F83W,F84M	UUU->TGG, UUC->ATG	998	58	16
15-67	F83W,F84L	UUU->TGG, UUC->TTG	1887	46	30
15-70	F83W,F84L	UUU->TGG, UUC->CTG	1723	50	27
15-73	F83W,F84M	UUU->TGG, UUC->ATG	1527	41	24
15-74	F83W,F84L	UUU->TGG, UUC->CTG	873	54	14
15-77*	F83W,F84W	UUU->TGG, UUC->TGG	5246	66	84
15-81	F83W,F84L	UUU->TGG, UUC->TTA	1027	37	16
15-86	F83W,F84M	UUU->TGG, UUC->ATG	1083	43	17
16-62	F100W	UUC->TGG	551	31	9
16-87*	F100Y	UUC->TAT	2574	38	41
16-90	F100Y	UUC->TAC	1663	41	26
17-10	F114V	UUU->GTG	767	39	12
17-11	F114V	UUU->GTA	864	33	14
17-13	F114I	UUU->ATA	1451	37	23
17-14	F114L	UUU->TTA	1729	47	28
27-106	F114K	UUU->AAG	116	23	2
27-121	F114W	UUU->TGG	1336	74	21
27-122*	F114M	UUU->ATG	3512	129	56
27-123	F114W	UUU->TGG	1480	73	24
27-124	F114W	UUU->TGG	1342	66	21
27-125	F114W	UUU->TGG	1372	65	22
27-126	F114Y	UUU->TAT	1362	83	22
18-15	F130L	UUU->CTG	1731	41	28
18-16	F130L	UUU->TTG	866	34	14
18-17	F130I	UUU->ATA	1624	38	26
18-18*	F130M	UUU->ATG	1652	37	26
18-19	I128M,F130L	ATT->ATG, UUU->CTG	1386	35	22
28-96	F130L	UUU->CTG	1884	88	30
19-20	F165L	UUC->CTA	189	17	3
19-21	F165L	UUC->TTG	220	17	4
19-22	F165M	UUC->ATG	812	35	13
19-24	F165W	UUC->TGG	398	29	6
29-107	F165T	UUC->ACG	122	22	2
29-108	F165A	UUC->GCA	974	57	16
29-109*	F165A	UUC->GCG	1070	51	17
29-110	F165Y	UUC->TAT	320	31	5
29-111	F165A	UUC->GCT	868	54	14
20-27	F223G	UUU->GGG	479	28	8
20-30	F223A	UUU->GCA	1010	35	16
20-31	F223M	UUU->ATG	3232	56	51
20-32	F223A	UUU->GCG	1945	17	31
20-48	F223V	UUU->GTA	3890	67	62
30-98*	F223T	UUU->ACG	4866	147	77
30-101	F223G	UUU->GGG	258	30	4
30-103	F223S	UUU->AGT	2158	79	34
