# Supplementary material for: Protein Evolution via Amino Acid and Codon Elimination
Source: PLoS One. 2010 Apr 26;5(4):e10104. doi: 10.1371/journal.pone.0010104 (PMC2859931; doi:10.1371/journal.pone.0010104)
Supplement: Table S2 — Oligonucleotides used in this study. (0.17 MB DOC) [file pone.0010104.s003.doc]

| Oligo name | Function | Sequence 5’-3’ |
| --- | --- | --- |
| otb134 | PCR primer for removal of *Nde*I site at 1575 in p335 (catatg → cacatg) | gttatccggatcacatgaaacggcatgac |
| otb135 | PCR primer for removal of *Bam*HI site at 1865 in p335 (ggatcc → ggttcc) | caacattgaagatggttccgttcaactagcag |
| otb136 | PCR primer for removal of *Bam*HI site at 2085 in p335 (ggatcc → ggttcc) | ctcggtacccggggttcctctagagtcg |
| otb137 | PCR primer for removal of *Nde*I site at 4928 in p335 (catatg → cacatg) | gtatttcacaccgcacatggtgcactctc |
| otb141 | Reverse PCR primer for amplification of the GFP gene | catcaggaattcattatttgtagagctcatcc |
| otb151 | Forward PCR primer for amplification of the GFP gene | cgtcaccatatggctagcaaaggagaagaacttttc |
| otb340 | Forward primer for insertion of NBR-library at F8 | NBRactggagttgtcccaattcttgttg |
| otb341 | Reverse primer for insertion of NBR-library at F8 | aagttcttctcctttgctagccatatgtatatc |
| otb342 | Forward primer for insertion of NBR-library at F41 | NBRtctgtcagtggagagggtgaaggtg |
| otb343 | Reverse primer for insertion of NBR-library at F41 | tttgtgcccattaacatcaccatctaattc |
| otb344 | Forward primer for insertion of NBR-library at F46 | NBRatttgcactactggaaaactacctgttcc |
| otb345 | Reverse primer for insertion of NBR-library at F46 | tttaagggtaagctttccgtatgtagcatc |
| otb346 | Forward primer for insertion of NBR-library at F71 | NBRtcccgttatccggatcacatgaaac |
| otb347 | Reverse primer for insertion of NBR-library at F71 | gcattgaacaccataagtcagagtagtgac |
| otb348 | Forward primer for insertion of NBR-library at F83,84 | NBRNBRaagagtgccatgcccgaagg |
| otb349 | Reverse primer for insertion of NBR-library at F83,84 | gtcatgccgtttcatgtgatccg |
| otb350 | Forward primer for insertion of NBR-library at F100 | NBRaaagatgacgggaactacaagacgc |
| otb351 | Reverse primer for insertion of NBR-library at F100 | agatatagtgcgttcctgtacataaccttcg |
| otb352 | Forward primer for insertion of NBR-library at F114 | NBRgaaggtgatacccttgttaatcgtatcgag |
| otb353 | Reverse primer for insertion of NBR-library at F114 | cttgacttcagcacgcgtcttgtag |
| otb354 | Forward primer for insertion of NBR-library at F130 | NBRaaagaagatggaaacattctcggacac |
| otb355 | Reverse primer for insertion of NBR-library at F130 | atcaataccttttaactcgatacgattaacaagg |
| otb356 | Forward primer for insertion of NBR-library at F165 | NBRaaaattcgccacaacattgaagatgg |
| otb357 | Reverse primer for insertion of NBR-library at F165 | gttagctttgattccattcttttgtttgtctg |
| otb358 | Forward primer for insertion of NBR-library at F223 | NBRgtaactgctgctgggattacacatgg |
| otb359 | Reverse primer for insertion of NBR-library at F223 | ctcaagaaggaccatgtggtcacg |
| otb412 | Multi Quick Change primer for re-introducing F8 | ggctagcaaaggagaagaactt**ttc**actggagttgtcccaattcttg |
| otb413 | Multi Quick Change primer for re-introducing F27 | gatgttaatgggcacaaa**ttt**tctgtcagtggagagggtgaagg |
| otb416 | Multi Quick Change primer for re-introducing F71 | gacttatggtgttcaatgc**ttt**tcccgttatccggatcacatg |
| otb419 | Multi Quick Change primer for re-introducing F130 | gttaaaaggtattgat**ttt**aaagaagatggaaacattctcggacacaaac |
| otb420 | Multi Quick Change primer for re-introducing F165 | gaatggaatcaaagctaac**ttc**aaaattcgccacaacattgaagatgg |
| otb434 | Oligo for assembly of 574-GFP (F8L) coding strand | tatggctagcaaaggagaagaacttctaactggagttgtcccaattcttgt |
| otb435 | Oligo for assembly of 574-GFP (F27L) coding strand | tgaattagatggtgatgttaatgggcacaaactgtctgtcagtggagagg |
| otb436 | Oligo for assembly of 574-GFP (F46A) coding strand | gtgaaggtgatgctacatacggaaagcttacccttaaagcgatttgcact |
| otb437 | Oligo for assembly of 574-GFP coding strand | actggaaaactacctgttccatggccaacacttgtcactactctgactta |
| otb438 | Oligo for assembly of 574-GFP (F71C) coding strand | tggtgttcaatgctgttcccgttatccggatcacatgaaacggcat |
| otb439 | Oligo for assembly of 574-GFP (F83W,F84W) coding strand | gactggtggaagagtgccatgcccgaaggttatgtacaggaacgcactatatct |
| otb440 | Oligo for assembly of 574-GFP (F100Y, F114M) coding strand | tataaagatgacgggaactacaagacgcgtgctgaagtcaagatggaagg |
| otb441 | Oligo for assembly of 574-GFP (F130M) coding strand | tgatacccttgttaatcgtatcgagttaaaaggtattgatatgaaagaag |
| otb442 | Oligo for assembly of 574-GFP coding strand | atggaaacattctcggacacaaactcgagtacaactataactcacacaat |
| otb443 | Oligo for assembly of 574-GFP (F165A) coding strand | gtatacatcacggcagacaaacaaaagaatggaatcaaagctaacgcgaa |
| otb444 | Oligo for assembly of 574-GFP coding strand | aattcgccacaacattgaagatggttccgttcaactagcagaccattatc |
| otb445 | Oligo for assembly of 574-GFP coding strand | aacaaaatactccaattggcgatggccctgtccttttaccagacaaccat |
| otb446 | Oligo for assembly of 574-GFP coding strand | tacctgtcgacacaatctgccctttcgaaagatcccaacgaaaagcgtga |
| otb447 | Oligo for assembly of 574-GFP (F223T) coding strand | ccacatggtccttcttgagacggtaactgctgctgggattacacatggca |
| otb448 | Oligo for assembly of 574-GFP coding strand | tggatgagctctacaaataatg |
| otb449 | Oligo for assembly of 574-GFP template strand | aattcattatttgtagagctcatccatgccatgtgtaatcccagca |
| otb450 | Oligo for assembly of 574-GFP (F223T) template strand | gcagttaccgtctcaagaaggaccatgtggtcacgcttttcgttgggatc |
| otb451 | Oligo for assembly of 574-GFP template strand | tttcgaaagggcagattgtgtcgacaggtaatggttgtctggtaaaagga |
| otb452 | Oligo for assembly of 574-GFP template strand | cagggccatcgccaattggagtattttgttgataatggtctgctagttga |
| otb453 | Oligo for assembly of 574-GFP (F165A) template strand | acggaaccatcttcaatgttgtggcgaattttcgcgttagctttgattcc |
| otb454 | Oligo for assembly of 574-GFP template strand | attcttttgtttgtctgccgtgatgtatacattgtgtgagttatagttgt |
| otb455 | Oligo for assembly of 574-GFP (F130M) template strand | actcgagtttgtgtccgagaatgtttccatcttctttcatatcaatacct |
| otb456 | Oligo for assembly of 574-GFP (F114M) template strand | tttaactcgatacgattaacaagggtatcaccttccatcttgacttcagc |
| otb457 | Oligo for assembly of 574-GFP (F100Y) template strand | acgcgtcttgtagttcccgtcatctttataagatatagtgcgttcctgta |
| otb458 | Oligo for assembly of 574-GFP (F83W,F84W) template strand | cataaccttcgggcatggcactcttccaccagtcatgccgtttcatgtga |
| otb459 | Oligo for assembly of 574-GFP (F71C) template strand | tccggataacgggaacagcattgaacaccataagtcagagtagtgacaag |
| otb460 | Oligo for assembly of 574-GFP (F46A) template strand | tgttggccatggaacaggtagttttccagtagtgcaaatcgctttaaggg |
| otb461 | Oligo for assembly of 574-GFP (F27L) template strand | taagctttccgtatgtagcatcaccttcaccctctccactgacagacagt |
| otb462 | Oligo for assembly of 574-GFP template strand | ttgtgcccattaacatcaccatctaattcaacaagaattgggacaactcc |
| otb463 | Oligo for assembly of 574-GFP (F8L) template strand | agttagaagttcttctcctttgctagcca |
| otb464 | Oligo for introduction of NVN-codon at F8 in GFP coding strand | tatggctagcaaaggagaagaacttnvnactggagttgtcccaattcttgt |
| otb465 | Oligo for introduction of NBR-codon at F8 in GFP coding strand | tatggctagcaaaggagaagaacttnbractggagttgtcccaattcttgt |
| otb466 | Oligo for introduction of NBR-codon at F27 in GFP coding strand | tgaattagatggtgatgttaatgggcacaaanbrtctgtcagtggagagg |
| otb467 | Oligo for introduction of NVN-codon at F27 in GFP coding strand | tgaattagatggtgatgttaatgggcacaaanvntctgtcagtggagagg |
| otb468 | Oligo for introduction of NBR-codon at F114 in GFP coding strand | tataaagatgacgggaactacaagacgcgtgctgaagtcaagnbrgaagg |
| otb469 | Oligo for introduction of NVN-codon at F114 in GFP coding strand | tataaagatgacgggaactacaagacgcgtgctgaagtcaagnvngaagg |
| otb470 | Oligo for introduction of NBR-codon at F8 in GFP template strand | agtyvnaagttcttctcctttgctagccata |
| otb471 | Oligo for introduction of NVN-codon at F8 in GFP template strand | agtnbnaagttcttctcctttgctagccata |
| otb472 | Oligo for introduction of NBR-codon at F27 in GFP template strand | taagctttccgtatgtagcatcaccttcaccctctccactgacagayvnt |
| otb473 | Oligo for introduction of NVN-codon at F27 in GFP template strand | taagctttccgtatgtagcatcaccttcaccctctccactgacaganbnt |
| otb474 | Oligo for introduction of NBR-codon at F114 in GFP template strand | tttaactcgatacgattaacaagggtatcaccttcyvncttgacttcagc |
| otb475 | Oligo for introduction of NVN-codon at F114 in GFP template strand | tttaactcgatacgattaacaagggtatcaccttcnbncttgacttcagc |
| otb477 | Oligo for reintroduction of F130 in 574-GFP coding strand | tgatacccttgttaatcgtatcgagttaaaaggtattgattttaaagaag |
| otb478 | Oligo for reintroduction of F165 in 574-GFP coding strand | gtatacatcacggcagacaaacaaaagaatggaatcaaagctaacttcaa |
| otb480 | Oligo for reintroduction of F130 in 574-GFP template strand | actcgagtttgtgtccgagaatgtttccatcttctttaaaatcaatacct |
| otb481 | Oligo for reintroduction of F165 in 574-GFP template strand | acggaaccatcttcaatgttgtggcgaattttgaagttagctttgattcc |
| otb482 | Oligo for reintroduction of F71 in 574-GFP coding strand | tggtgttcaatgcttttcccgttatccggatcacatgaaacggcat |
| otb483 | Oligo for reintroduction of F71 in 574-GFP template strand | tccggataacgggaaaagcattgaacaccataagtcagagtagtgacaag |
| otb509 | Oligo for reintroduction of F8 in 574-GFP coding strand | tatggctagcaaaggagaagaacttttcactggagttgtcccaattcttgt |
| otb510 | Oligo for reintroduction of F8 in 574-GFP template strand | agtgaaaagttcttctcctttgctagcca |
| otb511 | Oligo for reintroduction of F27 in 574-GFP coding strand | tgaattagatggtgatgttaatgggcacaaattttctgtcagtggagagg |
| otb512 | Oligo for reintroduction of F27 in 574-GFP template strand | taagctttccgtatgtagcatcaccttcaccctctccactgacagaaaat |
| otb513 | Oligo for introduction of NBR-codon at F71 in GFP coding strand | tggtgttcaatgcNBRtcccgttatccggatcacatgaaacggcat |
| otb514 | Oligo for introduction of NBR-codon at F71 in GFP template strand | tccggataacgggaYVNgcattgaacaccataagtcagagtagtgacaag |
| otb515 | Oligo for introduction of NBR-codon at F71 in GFP coding strand | tgatacccttgttaatcgtatcgagttaaaaggtattgatNBRaaagaag |
| otb516 | Oligo for introduction of NBR-codon at F71 in GFP template strand | actcgagtttgtgtccgagaatgtttccatcttctttYVNatcaatacct |
| otb517 | Oligo for introduction of NBR-codon at F165 in GFP coding strand | gtatacatcacggcagacaaacaaaagaatggaatcaaagctaacNBRaa |
| otb518 | Oligo for introduction of NBR-codon at F165 in GFP template strand | acggaaccatcttcaatgttgtggcgaattttYVNgttagctttgattcc |
| otb519 | Oligo for introduction of NVN-codon at F71 in GFP coding strand | tggtgttcaatgcNVNtcccgttatccggatcacatgaaacggcat |
| otb520 | Oligo for introduction of NVN-codon at F71 in GFP template strand | tccggataacgggaNBNgcattgaacaccataagtcagagtagtgacaag |
| otb521 | Oligo for introduction of NVN-codon at F130 in GFP coding strand | tgatacccttgttaatcgtatcgagttaaaaggtattgatNVNaaagaag |
| otb522 | Oligo for introduction of NVN-codon at F130 in GFP template strand | actcgagtttgtgtccgagaatgtttccatcttctttNBNatcaatacct |
| otb523 | Oligo for introduction of NVN-codon at F165 in GFP coding strand | gtatacatcacggcagacaaacaaaagaatggaatcaaagctaacNVNaa |
| otb524 | Oligo for introduction of NVN-codon at F165 in GFP template strand | acggaaccatcttcaatgttgtggcgaattttNBNgttagctttgattcc |
| otb525 | Oligo for introduction of TTA-codon at F71 in GFP coding strand | tggtgttcaatgcttatcccgttatccggatcacatgaaacggcat |
| otb526 | Oligo for introduction of TTA-codon at F71 in GFP template strand | tccggataacgggataagcattgaacaccataagtcagagtagtgacaag |
| otb527 | Oligo for introduction of TTA-codon at F130 in GFP coding strand | tgatacccttgttaatcgtatcgagttaaaaggtattgatttaaaagaag |
| otb528 | Oligo for introduction of TTA-codon at F130 in GFP template strand | actcgagtttgtgtccgagaatgtttccatcttcttttaaatcaatacct |
| otb529 | Oligo for introduction of TTG-codon at F8 in GFP coding strand | tatggctagcaaaggagaagaacttttgactggagttgtcccaattcttgt |
| otb530 | Oligo for introduction of TTG-codon at F8 in GFP template strand | agtcaaaagttcttctcctttgctagccata |
| otb531 | Oligo for introduction of TTA-codon at F114 in GFP coding strand | tataaagatgacgggaactacaagacgcgtgctgaagtcaagttagaagg |
| otb532 | Oligo for introduction of TTA-codon at F114 in GFP template strand | tttaactcgatacgattaacaagggtatcaccttctaacttgacttcagc |
| otb533 | Oligo for introduction of TGG-codon at F114 in GFP coding strand | tataaagatgacgggaactacaagacgcgtgctgaagtcaagtgggaagg |
| otb534 | Oligo for introduction of TGG-codon at F114 in GFP template strand | tttaactcgatacgattaacaagggtatcaccttcccacttgacttcagc |
| otb535 | Oligo for introduction of GTA-codon at F165 in GFP coding strand | gtatacatcacggcagacaaacaaaagaatggaatcaaagctaacgtaaa |
| otb536 | Oligo for introduction of GTA-codon at F165 in GFP template strand | acggaaccatcttcaatgttgtggcgaatttttacgttagctttgattcc |
| otb539 | Oligo for introduction of NBR-codon at F27 and CGT-codon at S30 in GFP coding strand | tgaattagatggtgatgttaatgggcacaaaNBRtctgtccgtggagagg |
| otb540 | Oligo for introduction of NVN-codon at F27 and CGT-codon at S30 in GFP coding strand | tgaattagatggtgatgttaatgggcacaaaNVNtctgtccgtggagagg |
| otb541 | Oligo for introduction of AAC-codon at Y39 (+F46A) in GFP coding strand | gtgaaggtgatgctacaaacggaaagcttacccttaaagcgatttgcact |
| otb542 | Oligo for introduction of ACC-codon at N105 (+F100Y and F114M) in GFP coding strand | tataaagatgacgggacctacaagacgcgtgctgaagtcaagatggaagg |
| otb543 | Oligo for introduction of ATT-codon at F130 in GFP coding strand | tgatacccttgttaatcgtatcgagttaaaaggtattgatattaaagaag |
| otb544 | Oligo for introduction of GTG-codon at F130 in GFP coding strand | tgatacccttgttaatcgtatcgagttaaaaggtattgatgtgaaagaag |
| otb545 | Oligo for introduction of GTT-codon at I171 in GFP coding strand | aattcgccacaacgttgaagatggttccgttcaactagcagaccattatc |
| otb546 | Oligo for introduction of GTT-codon at A206 in GFP coding strand | tacctgtcgacacaatctgttctttcgaaagatcccaacgaaaagcgtga |
| otb547 | Oligo for introduction of GTT-codon at A206 in GFP template strand | tttcgaaagaacagattgtgtcgacaggtaatggttgtctggtaaaagga |
| otb548 | Oligo for introduction of NBR-library at F165 and GTT-codon at I171 in GFP template strand | acggaaccatcttcaacgttgtggcgaattttYVNgttagctttgattcc |
| otb549 | Oligo for introduction of NVN-library at F165 and GTT-codon at I171 in GFP template strand | acggaaccatcttcaacgttgtggcgaattttNBNgttagctttgattcc |
| otb550 | Oligo for introduction of ATT-codon at F130 in GFP template strand | actcgagtttgtgtccgagaatgtttccatcttctttaatatcaatacct |
| otb551 | Oligo for introduction of GTG-codon at F130 in GFP template strand | actcgagtttgtgtccgagaatgtttccatcttctttcacatcaatacct |
| otb552 | Oligo for introduction of ACC-codon at N105 (+Y100) in GFP template strand | acgcgtcttgtaggtcccgtcatctttataagatatagtgcgttcctgta |
| otb553 | Oligo for introduction of NBR-library at F27 and ACC-codon at Y39 and CGT-codon at S30 in GFP template strand | taagctttccgtttgtagcatcaccttcaccctctccacggacagaYVNt |
| otb554 | Oligo for introduction of NVN-library at F27 and ACC-codon at Y39 and CGT-codon at S30 in GFP template strand | taagctttccgtttgtagcatcaccttcaccctctccacggacagaNBNt |
| otb558 | Forward PCR primer for introduction of His6 at the N-terminus of GFP | cgtcaccatatgcatcaccatcaccatcacgctagcaaaggagaagaacttttc |
