# Supplementary material for: Protein Evolution via Amino Acid and Codon Elimination
Source: PLoS One. 2010 Apr 26;5(4):e10104. doi: 10.1371/journal.pone.0010104 (PMC2859931; doi:10.1371/journal.pone.0010104)
Supplement: Table S3 — Oligonucleotide combinations for construction of GFPs with reduced Phe content by gene assembly. The numbers indicated for forward (column 1) and reverse (column 2) oligonucleotides are defined in Table S2. “Phe-residue” in column 3 indicates which Phe-codon(s) in GFP-Ref. that is covered by the oligonucleotide in question. The (−;−) notation signifies forward (left dash) and reverse (right dash) oligonucleotide. The column entitled “substitution” states whether the given oligonucleotide contains the original Phe-codon or a substitution. See Materials and Methods for details. (0.41 MB DOC) [file pone.0010104.s004.doc]

| **p574-c20** |  |  |  |  |
| --- | --- | --- | --- | --- |
|  |  |  |  |  |
|  | forward | reverse | phe-residue | substitution |
|  |  |  |  |  |
|  | 434 | 463 | (8;8) | (L;L) |
|  | 435 | 462 | (27;-) | (L;-) |
|  | 436 | 461 | (46;27) | (A;L) |
|  | 437 | 460 | (-;46) | (-;A) |
|  | 438 | 459 | (71;71) | (C;C) |
|  | 439 | 458 | (83,84;83,84) | (W,W;W,W) |
|  | 440 | 457 | (100,114;100) | (Y,M;Y) |
|  | 441 | 456 | (130;114) | (M;M) |
|  | 442 | 455 | (-;130) | (-;M) |
|  | 443 | 454 | (165;-) | (A;-) |
|  | 444 | 453 | (-;165) | (-;A) |
|  | 445 | 452 | (-;-) | (-;-) |
|  | 446 | 451 | (-;-) | (-;-) |
|  | 447 | 450 | (223;223) | (T;T) |
|  | 448 | 449 | (-;-) | (-;-) |
|  |  |  |  |  |
| **F3-GFP libraries** |  |  |  |  |
| Phe: | 8,27,130 |  |  |  |
| library: | 71,165 |  |  |  |
|  | forward | reverse | phe-residue | substitution |
|  |  |  |  |  |
|  | 509 | 510 | (8;8) | (phe;phe) |
|  | 511 | 462 | (27;-) | (phe;-) |
|  | 436 | 512 | (46;27) | (A;phe) |
|  | 437 | 460 | (-;46) | (-;A) |
|  | 513,519 | 514,520 | (71;71) | (library;library) |
|  | 439 | 458 | (83,84;83,84) | (W,W;W,W) |
|  | 440 | 457 | (100,114;100) | (Y,M;Y) |
|  | 477 | 456 | (130;114) | (phe;M) |
|  | 442 | 480 | (-;130) | (-;phe) |
|  | 517,523 | 454 | (165;-) | (library;-) |
|  | 444 | 518,524 | (-;165) | (-;library) |
|  | 445 | 452 | (-;-) | (-;-) |
|  | 446 | 451 | (-;-) | (-;-) |
|  | 447 | 450 | (223;223) | (T;T) |
|  | 448 | 449 | (-;-) | (-;-) |
|  |  |  |  |  |
|  |  |  |  |  |
| Phe: | 27,130,165 |  |  |  |
| library: | 8,71,114 |  |  |  |
|  | forward | reverse | phe-residue | substitution |
|  |  |  |  |  |
|  | 464,465 | 470,471 | (8;8) | (library;library) |
|  | 511 | 462 | (27;-) | (phe;-) |
|  | 436 | 512 | (46;27) | (A;phe) |
|  | 437 | 460 | (-;46) | (-;A) |
|  | 513,519 | 514,520 | (71;71) | (library;library) |
|  | 439 | 458 | (83,84;83,84) | (W,W;W,W) |
|  | 468,469 | 457 | (100,114;100) | (Y,library;Y) |
|  | 477 | 474,475 | (130;114) | (phe;library) |
|  | 442 | 480 | (-;130) | (-;phe) |
|  | 478 | 454 | (165;-) | (phe;-) |
|  | 444 | 481 | (-;165) | (-;phe) |
|  | 445 | 452 | (-;-) | (-;-) |
|  | 446 | 451 | (-;-) | (-;-) |
|  | 447 | 450 | (223;223) | (T;T) |
|  | 448 | 449 | (-;-) | (-;-) |
| Phe: | 8,27,71 |  |  |  |
| library: | 130,165 |  |  |  |
|  | forward | reverse | phe-residue | substitution |
|  |  |  |  |  |
|  | 509 | 510 | (8;8) | (phe;phe) |
|  | 511 | 462 | (27;-) | (phe;-) |
|  | 436 | 512 | (46;27) | (A;phe) |
|  | 437 | 460 | (-;46) | (-;A) |
|  | 482 | 483 | (71;71) | (phe;phe) |
|  | 439 | 458 | (83,84;83,84) | (W,W;W,W) |
|  | 440 | 457 | (100,114;100) | (Y,M;Y) |
|  | 515,521 | 456 | (130;114) | (library;M) |
|  | 442 | 516,522 | (-;130) | (-;library) |
|  | 517,523 | 454 | (165;-) | (library;-) |
|  | 444 | 518,524 | (-;165) | (-;library) |
|  | 445 | 452 | (-;-) | (-;-) |
|  | 446 | 451 | (-;-) | (-;-) |
|  | 447 | 450 | (223;223) | (T;T) |
|  | 448 | 449 | (-;-) | (-;-) |
|  |  |  |  |  |
|  |  |  |  |  |
| Phe: | 8,27,165 |  |  |  |
| library: | 71,130 |  |  |  |
|  | forward | reverse | phe-residue | substitution |
|  |  |  |  |  |
|  | 509 | 510 | (8;8) | (phe;phe) |
|  | 511 | 462 | (27;-) | (phe;-) |
|  | 436 | 512 | (46;27) | (A;phe) |
|  | 437 | 460 | (-;46) | (-;A) |
|  | 513,519 | 514,520 | (71;71) | (library;library) |
|  | 439 | 458 | (83,84;83,84) | (W,W;W,W) |
|  | 440 | 457 | (100,114;100) | (Y,M;Y) |
|  | 515,521 | 456 | (130;114) | (library;M) |
|  | 442 | 516,522 | (-;130) | (-;library) |
|  | 478 | 454 | (165;-) | (phe;-) |
|  | 444 | 481 | (-;165) | (-;phe) |
|  | 445 | 452 | (-;-) | (-;-) |
|  | 446 | 451 | (-;-) | (-;-) |
|  | 447 | 450 | (223;223) | (T;T) |
|  | 448 | 449 | (-;-) | (-;-) |
|  |  |  |  |  |
|  |  |  |  |  |
| Phe: | 27,71,165 |  |  |  |
| library: | 8,130 |  |  |  |
|  | forward | reverse | phe-residue | substitution |
|  |  |  |  |  |
|  | 464,465 | 470,471 | (8;8) | (library;library) |
|  | 511 | 462 | (27;-) | (phe;-) |
|  | 436 | 512 | (46;27) | (A;phe) |
|  | 437 | 460 | (-;46) | (-;A) |
|  | 482 | 483 | (71;71) | (phe;phe) |
|  | 439 | 458 | (83,84;83,84) | (W,W;W,W) |
|  | 440 | 457 | (100,114;100) | (Y,M;Y) |
|  | 515,521 | 456 | (130;114) | (library;M) |
|  | 442 | 516,522 | (-;130) | (-;library) |
|  | 478 | 454 | (165;-) | (phe;-) |
|  | 444 | 481 | (-;165) | (-;phe) |
|  | 445 | 452 | (-;-) | (-;-) |
|  | 446 | 451 | (-;-) | (-;-) |
|  | 447 | 450 | (223;223) | (T;T) |
|  | 448 | 449 | (-;-) | (-;-) |
|  |  |  |  |  |
| Phe: | 27,71,130 |  |  |  |
| library: | 8,165 |  |  |  |
|  | forward | reverse | phe-residue | substitution |
|  |  |  |  |  |
|  | 464,465 | 470,471 | (8;8) | (library;library) |
|  | 511 | 462 | (27;-) | (phe;-) |
|  | 436 | 512 | (46;27) | (A;phe) |
|  | 437 | 460 | (-;46) | (-;A) |
|  | 482 | 483 | (71;71) | (phe;phe) |
|  | 439 | 458 | (83,84;83,84) | (W,W;W,W) |
|  | 440 | 457 | (100,114;100) | (Y,M;Y) |
|  | 477 | 456 | (130;114) | (phe;M) |
|  | 442 | 480 | (-;130) | (-;phe) |
|  | 517,523 | 454 | (165;-) | (library;-) |
|  | 444 | 518,524 | (-;165) | (-;library) |
|  | 445 | 452 | (-;-) | (-;-) |
|  | 446 | 451 | (-;-) | (-;-) |
|  | 447 | 450 | (223;223) | (T;T) |
|  | 448 | 449 | (-;-) | (-;-) |
|  |  |  |  |  |
|  |  |  |  |  |
| Phe: | 27,130,165 |  |  |  |
| library: | 8,71 |  |  |  |
|  | forward | reverse | phe-residue | substitution |
|  |  |  |  |  |
|  | 464,465 | 470,471 | (8;8) | (library;library) |
|  | 511 | 462 | (27;-) | (phe;-) |
|  | 436 | 512 | (46;27) | (A;phe) |
|  | 437 | 460 | (-;46) | (-;A) |
|  | 513,519 | 514,520 | (71;71) | (library;library) |
|  | 439 | 458 | (83,84;83,84) | (W,W;W,W) |
|  | 440 | 457 | (100,114;100) | (Y,M;Y) |
|  | 477 | 456 | (130;114) | (phe;M) |
|  | 442 | 480 | (-;130) | (-;phe) |
|  | 478 | 454 | (165;-) | (phe;-) |
|  | 444 | 481 | (-;165) | (-;phe) |
|  | 445 | 452 | (-;-) | (-;-) |
|  | 446 | 451 | (-;-) | (-;-) |
|  | 447 | 450 | (223;223) | (T;T) |
|  | 448 | 449 | (-;-) | (-;-) |
|  |  |  |  |  |
|  |  |  |  |  |
|  |  |  |  |  |
| Phe: | 8,71,130 |  |  |  |
| library: | 27,165 |  |  |  |
|  | forward | reverse | phe-residue | substitution |
|  |  |  |  |  |
|  | 509 | 510 | (8;8) | (phe;phe) |
|  | 466,467 | 462 | (27;-) | (library;-) |
|  | 436 | 472,473 | (46;27) | (A;library) |
|  | 437 | 460 | (-;46) | (-;A) |
|  | 482 | 483 | (71;71) | (phe;phe) |
|  | 439 | 458 | (83,84;83,84) | (W,W;W,W) |
|  | 440 | 457 | (100,114;100) | (Y,M;Y) |
|  | 477 | 456 | (130;114) | (phe;M) |
|  | 442 | 480 | (-;130) | (-;phe) |
|  | 517,523 | 454 | (165;-) | (library;-) |
|  | 444 | 518,524 | (-;165) | (-;library) |
|  | 445 | 452 | (-;-) | (-;-) |
|  | 446 | 451 | (-;-) | (-;-) |
|  | 447 | 450 | (223;223) | (T;T) |
|  | 448 | 449 | (-;-) | (-;-) |
| Phe: | 8,71,165 |  |  |  |
| library: | 27,130 |  |  |  |
|  | forward | reverse | phe-residue | substitution |
|  |  |  |  |  |
|  | 509 | 510 | (8;8) | (phe;phe) |
|  | 466,467 | 462 | (27;-) | (library;-) |
|  | 436 | 472,473 | (46;27) | (A;library) |
|  | 437 | 460 | (-;46) | (-;A) |
|  | 482 | 483 | (71;71) | (phe;phe) |
|  | 439 | 458 | (83,84;83,84) | (W,W;W,W) |
|  | 440 | 457 | (100,114;100) | (Y,M;Y) |
|  | 515,521 | 456 | (130;114) | (library;M) |
|  | 442 | 516,522 | (-;130) | (-;library) |
|  | 478 | 454 | (165;-) | (phe;-) |
|  | 444 | 481 | (-;165) | (-;phe) |
|  | 445 | 452 | (-;-) | (-;-) |
|  | 446 | 451 | (-;-) | (-;-) |
|  | 447 | 450 | (223;223) | (T;T) |
|  | 448 | 449 | (-;-) | (-;-) |
|  |  |  |  |  |
|  |  |  |  |  |
| Phe: | 8,130,165 |  |  |  |
| library: | 27,71 |  |  |  |
|  | forward | reverse | phe-residue | substitution |
|  |  |  |  |  |
|  | 509 | 510 | (8;8) | (phe;phe) |
|  | 466,467 | 462 | (27;-) | (library;-) |
|  | 436 | 472,473 | (46;27) | (A;library) |
|  | 437 | 460 | (-;46) | (-;A) |
|  | 513,519 | 514,520 | (71;71) | (library;library) |
|  | 439 | 458 | (83,84;83,84) | (W,W;W,W) |
|  | 440 | 457 | (100,114;100) | (Y,M;Y) |
|  | 477 | 456 | (130;114) | (phe;M) |
|  | 442 | 480 | (-;130) | (-;phe) |
|  | 478 | 454 | (165;-) | (phe;-) |
|  | 444 | 481 | (-;165) | (-;phe) |
|  | 445 | 452 | (-;-) | (-;-) |
|  | 446 | 451 | (-;-) | (-;-) |
|  | 447 | 450 | (223;223) | (T;T) |
|  | 448 | 449 | (-;-) | (-;-) |
|  |  |  |  |  |
|  |  |  |  |  |
| Phe: | 71,130,165 |  |  |  |
| library: | 8,27 |  |  |  |
|  | forward | reverse | phe-residue | substitution |
|  |  |  |  |  |
|  | 464,465 | 470,471 | (8;8) | (library;library) |
|  | 466,467 | 462 | (27;-) | (library;-) |
|  | 436 | 472,473 | (46;27) | (A;library) |
|  | 437 | 460 | (-;46) | (-;A) |
|  | 482 | 483 | (71;71) | (phe;phe) |
|  | 439 | 458 | (83,84;83,84) | (W,W;W,W) |
|  | 440 | 457 | (100,114;100) | (Y,M;Y) |
|  | 477 | 456 | (130;114) | (phe;M) |
|  | 442 | 480 | (-;130) | (-;phe) |
|  | 478 | 454 | (165;-) | (phe;-) |
|  | 444 | 481 | (-;165) | (-;phe) |
|  | 445 | 452 | (-;-) | (-;-) |
|  | 446 | 451 | (-;-) | (-;-) |
|  | 447 | 450 | (223;223) | (T;T) |
|  | 448 | 449 | (-;-) | (-;-) |
|  |  |  |  |  |
| **F0-GFP libraries** |  |  | S30R, Y39N, N105T, I171V, A206V |  |
| Library(130L): | 27,165 |  |  |  |
|  |  |  |  |  |
|  | forward | reverse | phe-residue | substitution |
|  |  |  |  |  |
|  | 529 | 530 | (8;8) | (leu;leu) |
|  | 539+540 | 462 | (27;-) | (library +S30R;-) |
|  | 541 | 553+554 | (46;27) | (ala +Y39N;library + Y39N + S30R) |
|  | 437 | 460 | (-;46) | (-;ala) |
|  | 525 | 526 | (71;71) | (leu;leu) |
|  | 439 | 458 | (83,84;83,84) | (W,W;W,W) |
|  | 542 | 552 | (100,114;100) | (Y,M + N105T;Y +N105T) |
|  | 527 | 456 | (130;114) | (Leu;M) |
|  | 442 | 528 | (-;130) | (-;Leu) |
|  | 517+523 | 454 | (165;-) | (library;-) |
|  | 545 | 548+549 | (-;165) | (I171V;library + I171V) |
|  | 445 | 452 | (-;-) | (-;-) |
|  | 546 | 547 | (-;-) | (A206V;A206V) |
|  | 447 | 450 | (223;223) | (T;T) |
|  | 448 | 449 | (-;-) | (-;-) |
|  |  |  |  |  |
| Library(130I): | 27,165 |  |  |  |
|  | forward | reverse | phe-residue | substitution |
|  |  |  |  |  |
|  | 529 | 530 | (8;8) | (leu;leu) |
|  | 539+540 | 462 | (27;-) | (library +S30R;-) |
|  | 541 | 553+554 | (46;27) | (ala +Y39N;library + Y39N + S30R) |
|  | 437 | 460 | (-;46) | (-;ala) |
|  | 525 | 526 | (71;71) | (leu;leu) |
|  | 439 | 458 | (83,84;83,84) | (W,W;W,W) |
|  | 542 | 552 | (100,114;100) | (Y,M + N105T;Y +N105T) |
|  | 543 | 456 | (130;114) | (ile;M) |
|  | 442 | 550 | (-;130) | (-;ile) |
|  | 517+523 | 454 | (165;-) | (library;-) |
|  | 545 | 548+549 | (-;165) | (I171V;library + I171V) |
|  | 445 | 452 | (-;-) | (-;-) |
|  | 546 | 547 | (-;-) | (A206V;A206V) |
|  | 447 | 450 | (223;223) | (T;T) |
|  | 448 | 449 | (-;-) | (-;-) |
|  |  |  |  |  |
| Library(130V): | 27,165 |  |  |  |
|  | forward | reverse | phe-residue | substitution |
|  |  |  |  |  |
|  | 529 | 530 | (8;8) | (leu;leu) |
|  | 539+540 | 462 | (27;-) | (library +S30R;-) |
|  | 541 | 553+554 | (46;27) | (ala +Y39N;library + Y39N + S30R) |
|  | 437 | 460 | (-;46) | (-;ala) |
|  | 525 | 526 | (71;71) | (leu;leu) |
|  | 439 | 458 | (83,84;83,84) | (W,W;W,W) |
|  | 542 | 552 | (100,114;100) | (Y,M + N105T;Y +N105T) |
|  | 544 | 456 | (130;114) | (Val;M) |
|  | 442 | 551 | (-;130) | (-;Val) |
|  | 517+523 | 454 | (165;-) | (library;-) |
|  | 545 | 548+549 | (-;165) | (I171V;library + I171V) |
|  | 445 | 452 | (-;-) | (-;-) |
|  | 546 | 547 | (-;-) | (A206V;A206V) |
|  | 447 | 450 | (223;223) | (T;T) |
|  | 448 | 449 | (-;-) | (-;-) |
