# Supplementary figures and images for: Protein Evolution via Amino Acid and Codon Elimination
Source: PLoS One. 2010 Apr 26;5(4):e10104. doi: 10.1371/journal.pone.0010104 (PMC2859931; doi:10.1371/journal.pone.0010104)

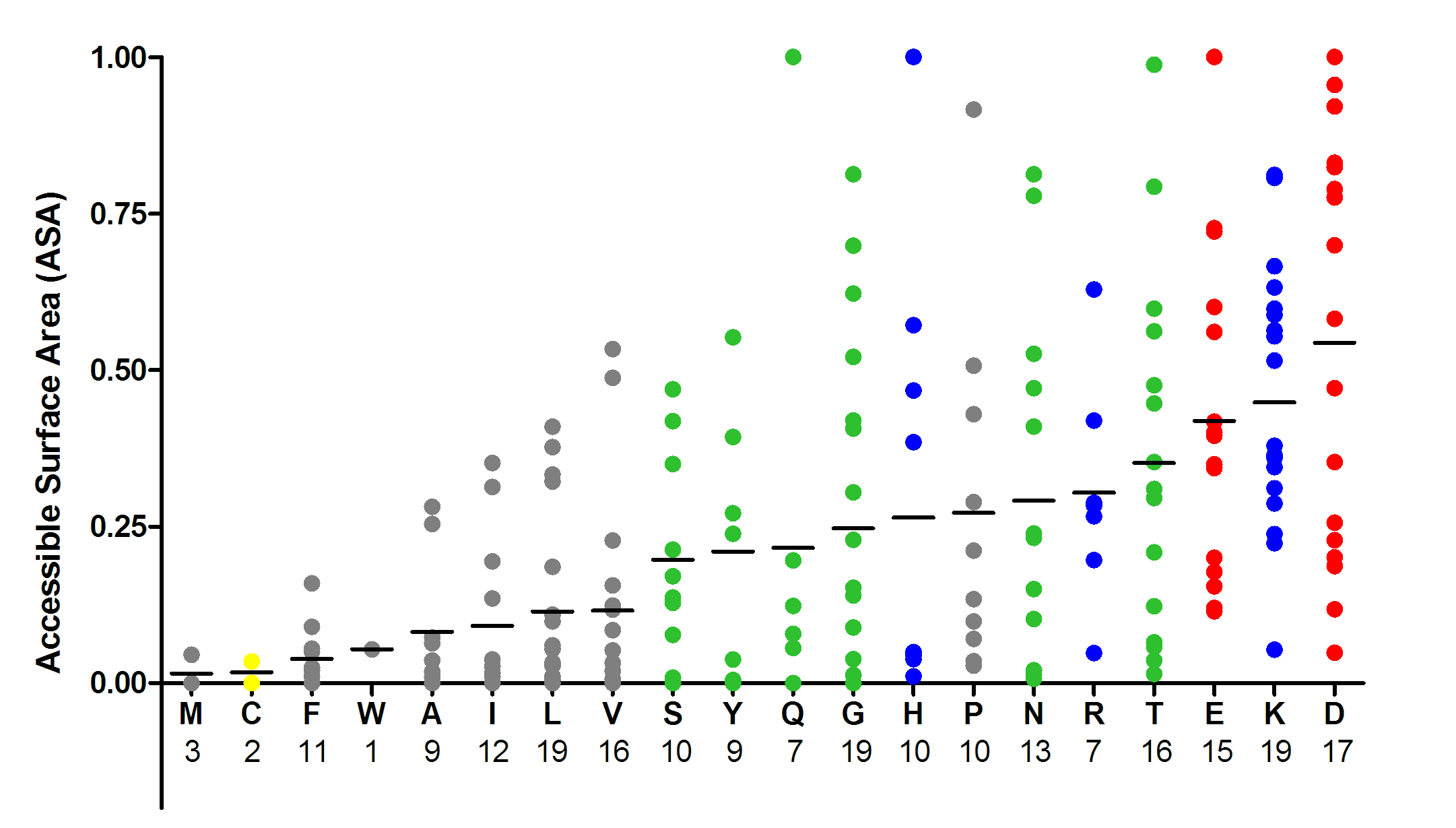

Supplement: Figure S1 — Amino acid solvent accessibility in GFP. Solvent accessibility analysis of amino acids in folding reporter GFP (PDB file 2B3Q) using ASAview software. The global count of each amino acid is given below the x-axis. Amino acid colour code: hydrophobic (grey), cystein (yellow), polar uncharged (green), positive (blue), and negative (red). (0.74 MB TIF) [file pone.0010104.s005.tif]

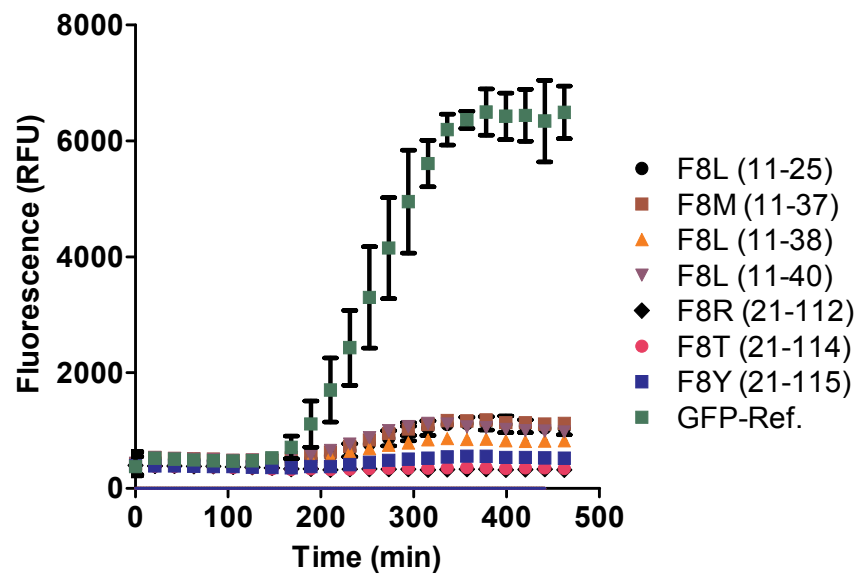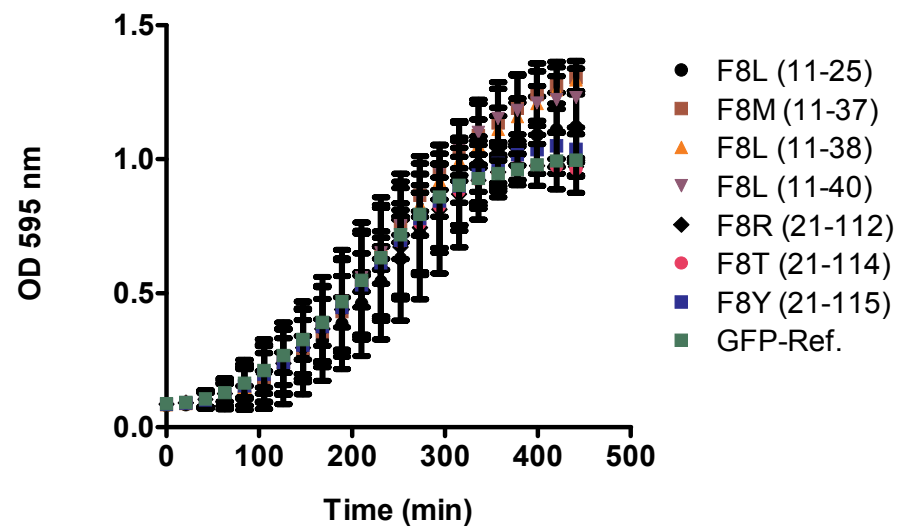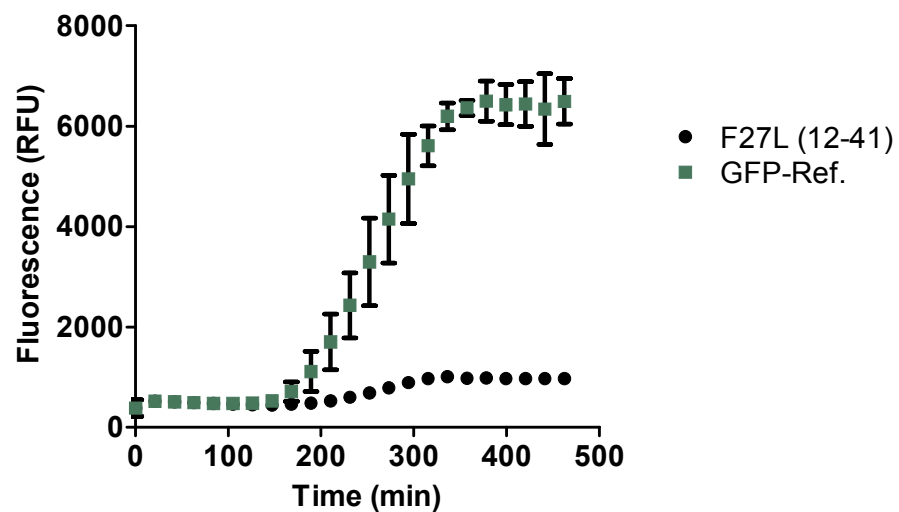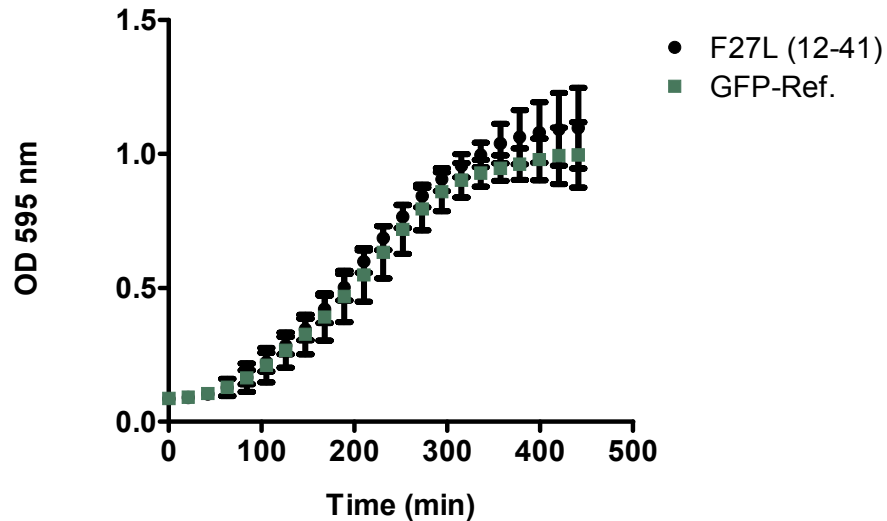

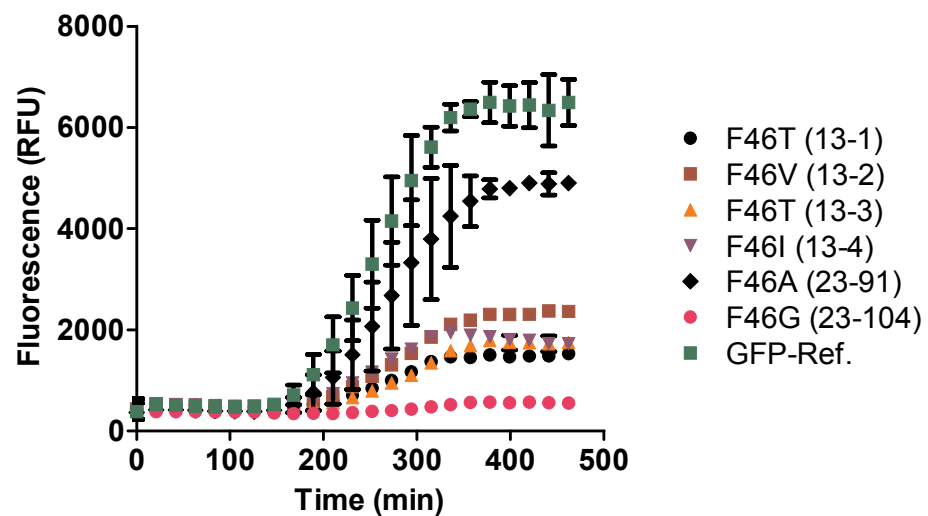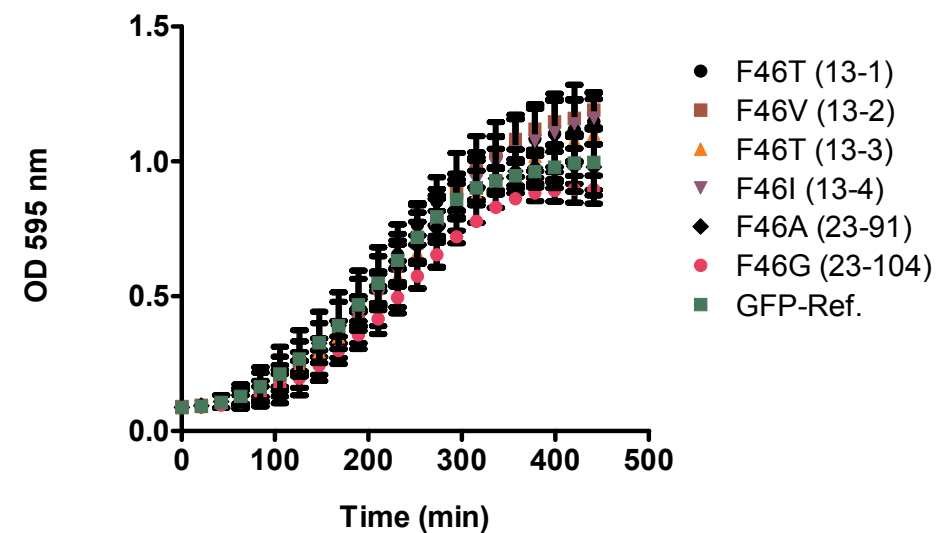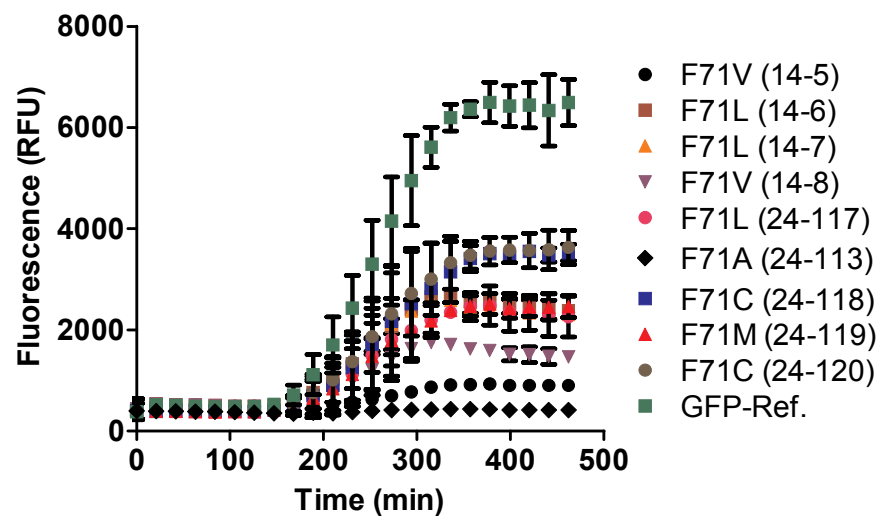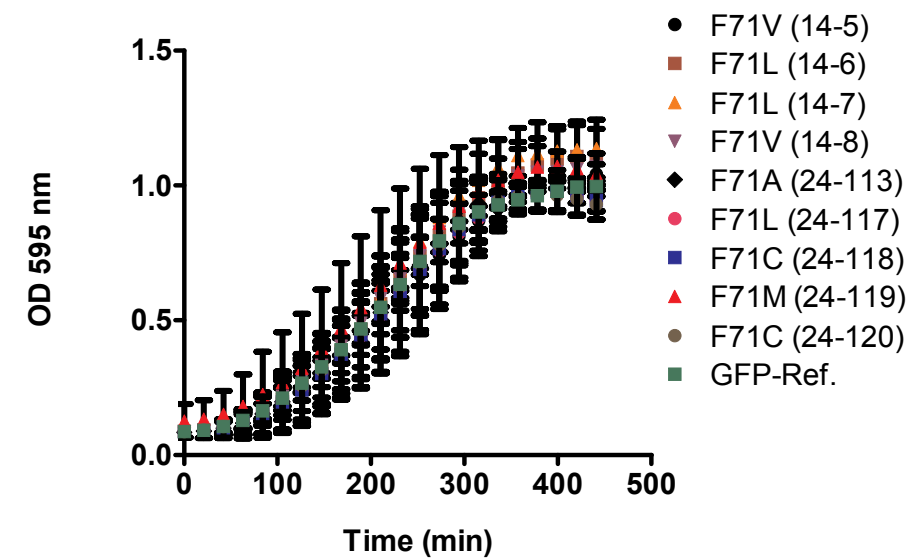

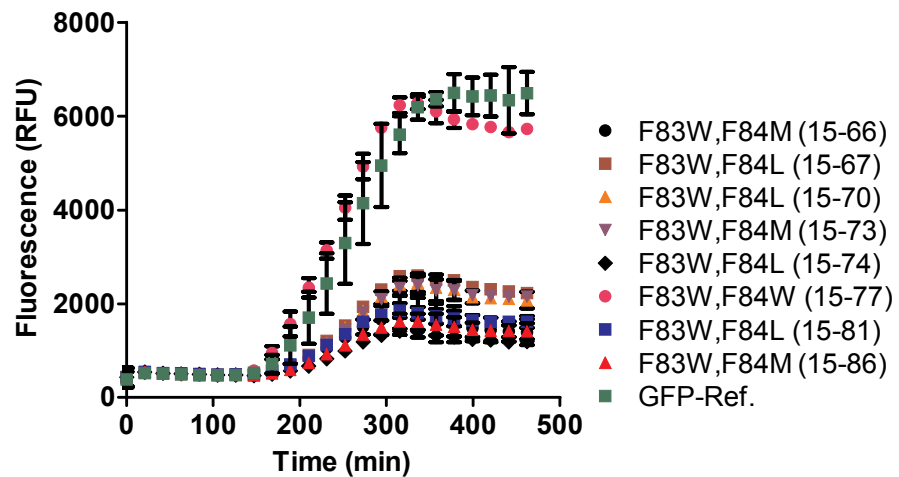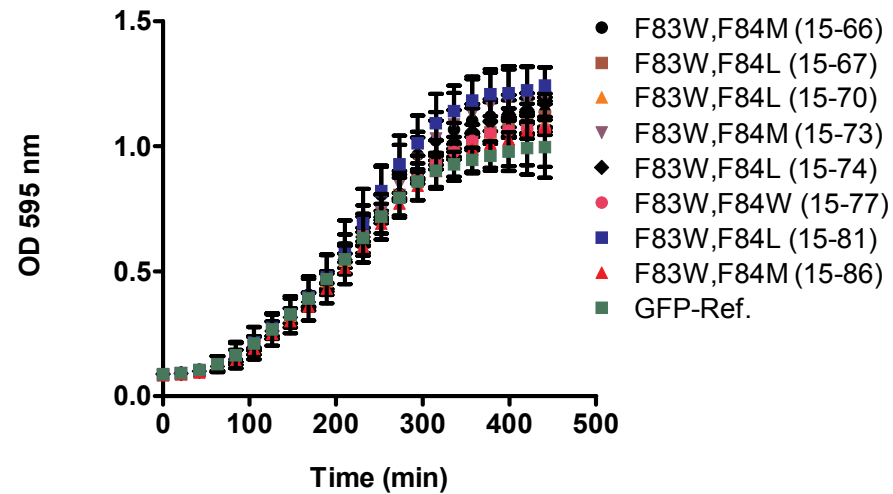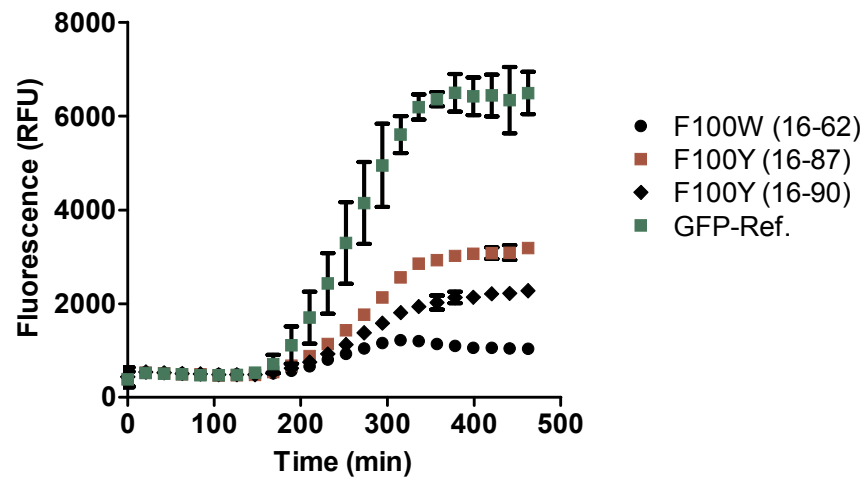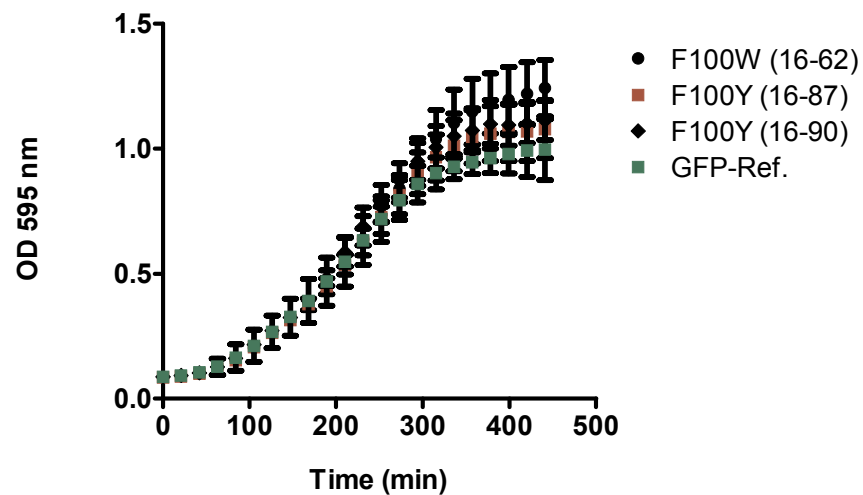

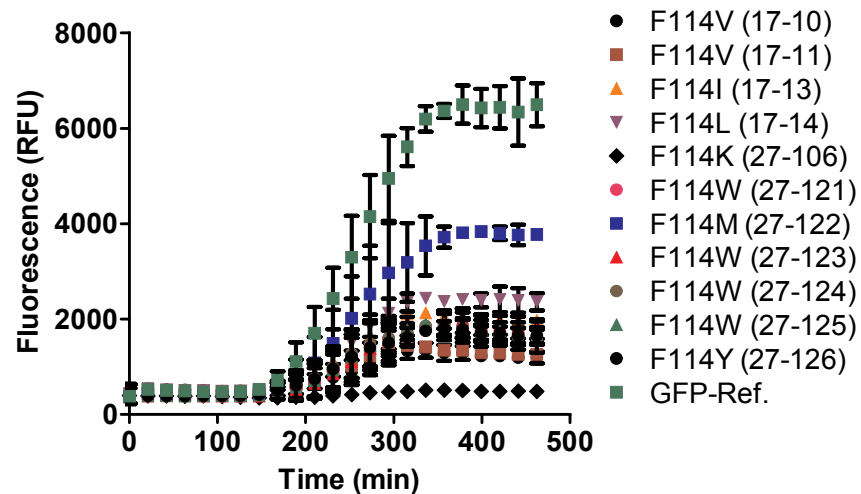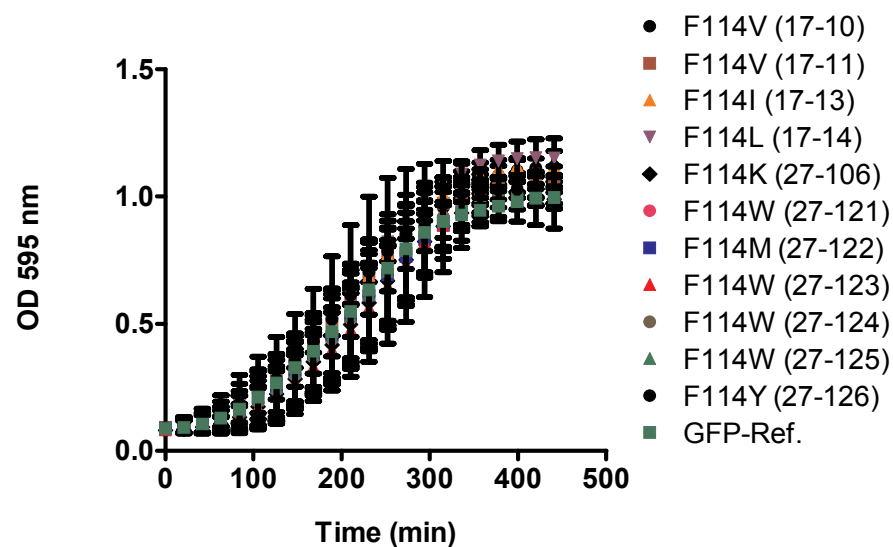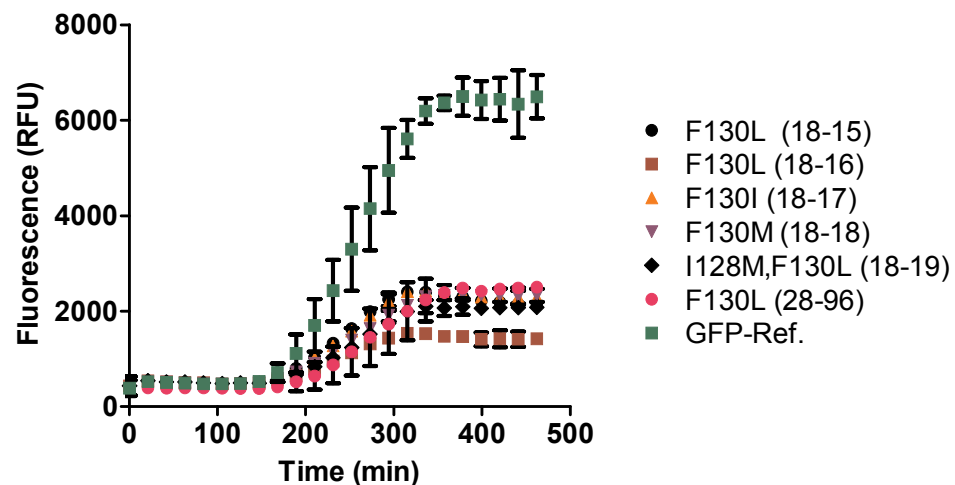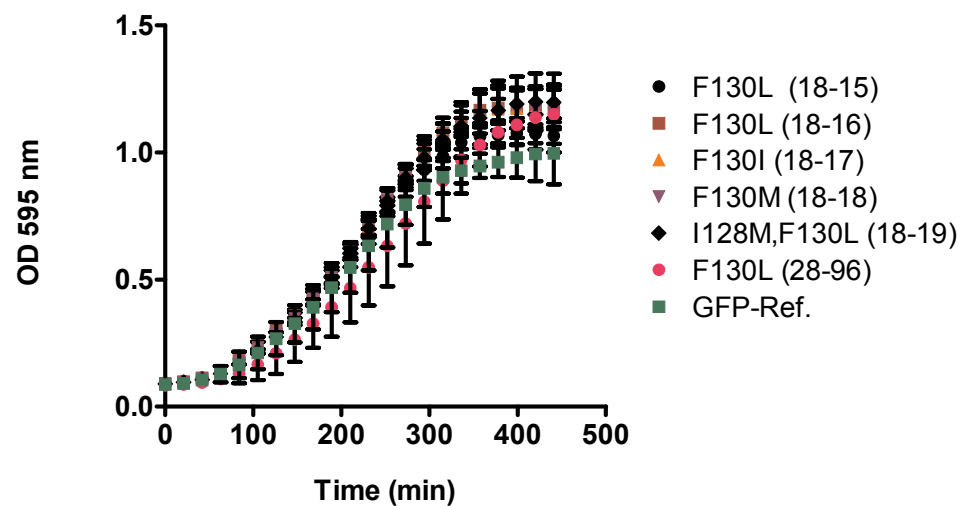

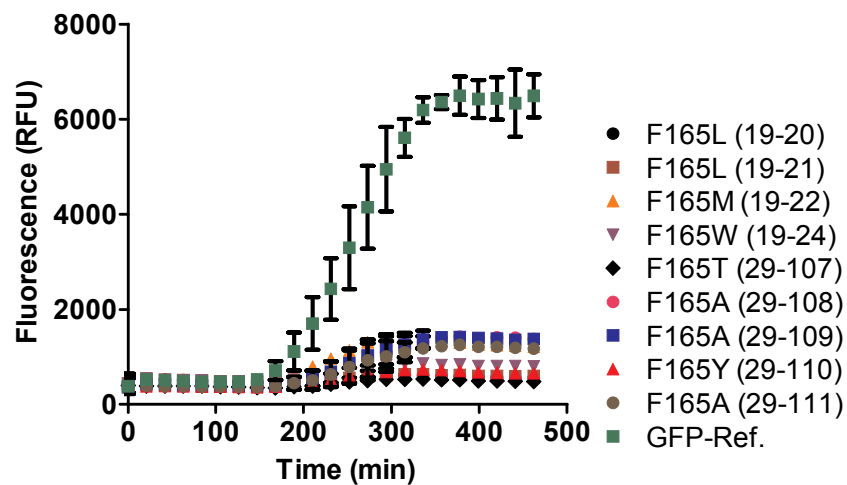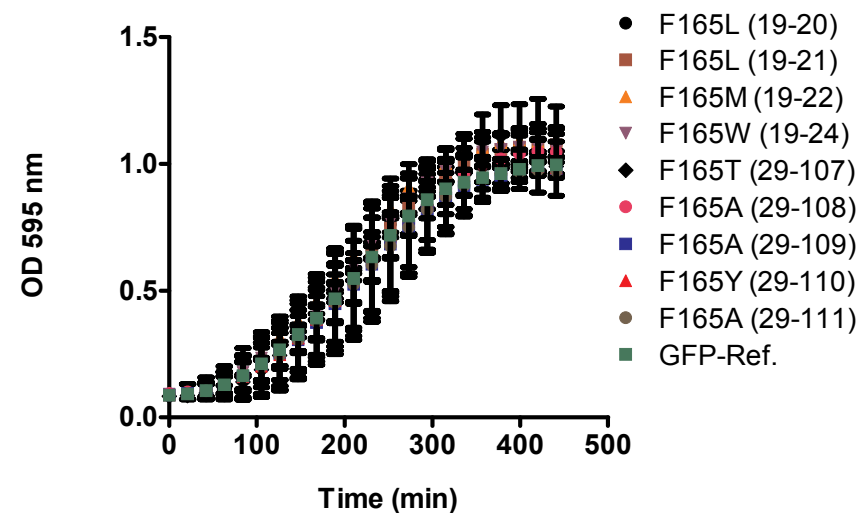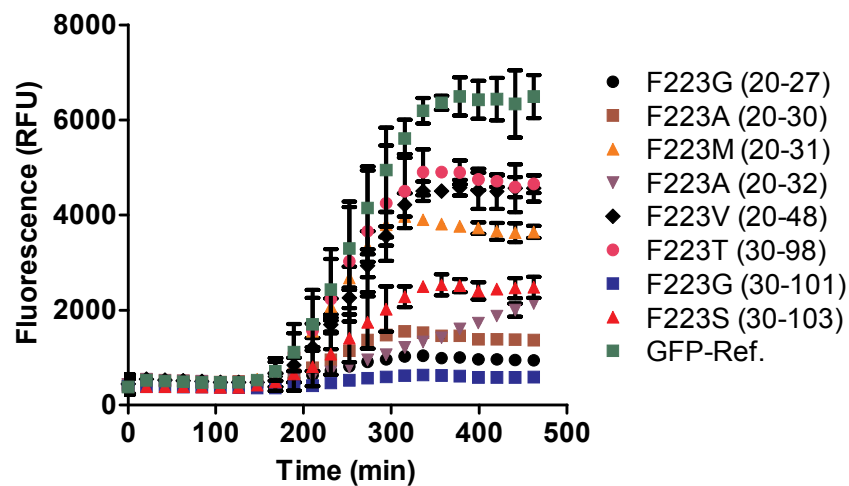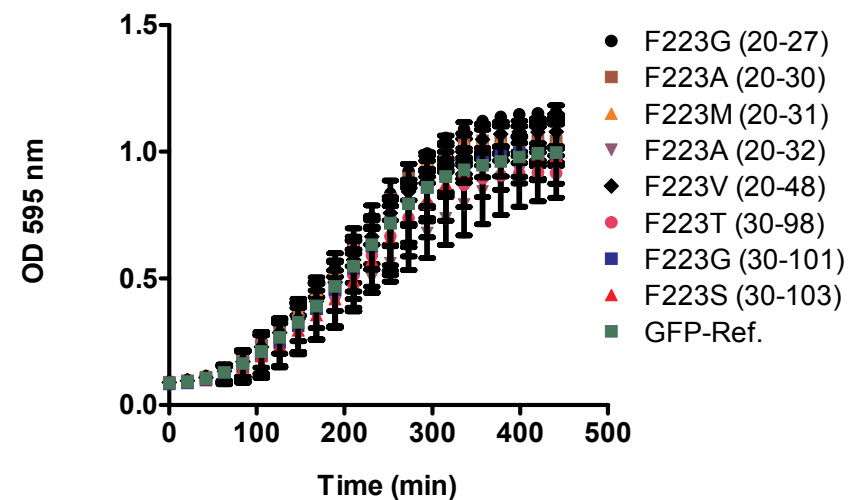

Supplement: Figure S2 — In vivo GFP fluorescence accumulation and growth curves for all single-substitution mutants analyzed. Overnight starter cultures were diluted 100-fold, into LB-amp supplemented with 0.1% arabinose and grown for 8 h at 37° C. All measurements were performed in duplicates and the mean and SD for each data point is shown. (0.19 MB PDF) [file pone.0010104.s006.pdf]

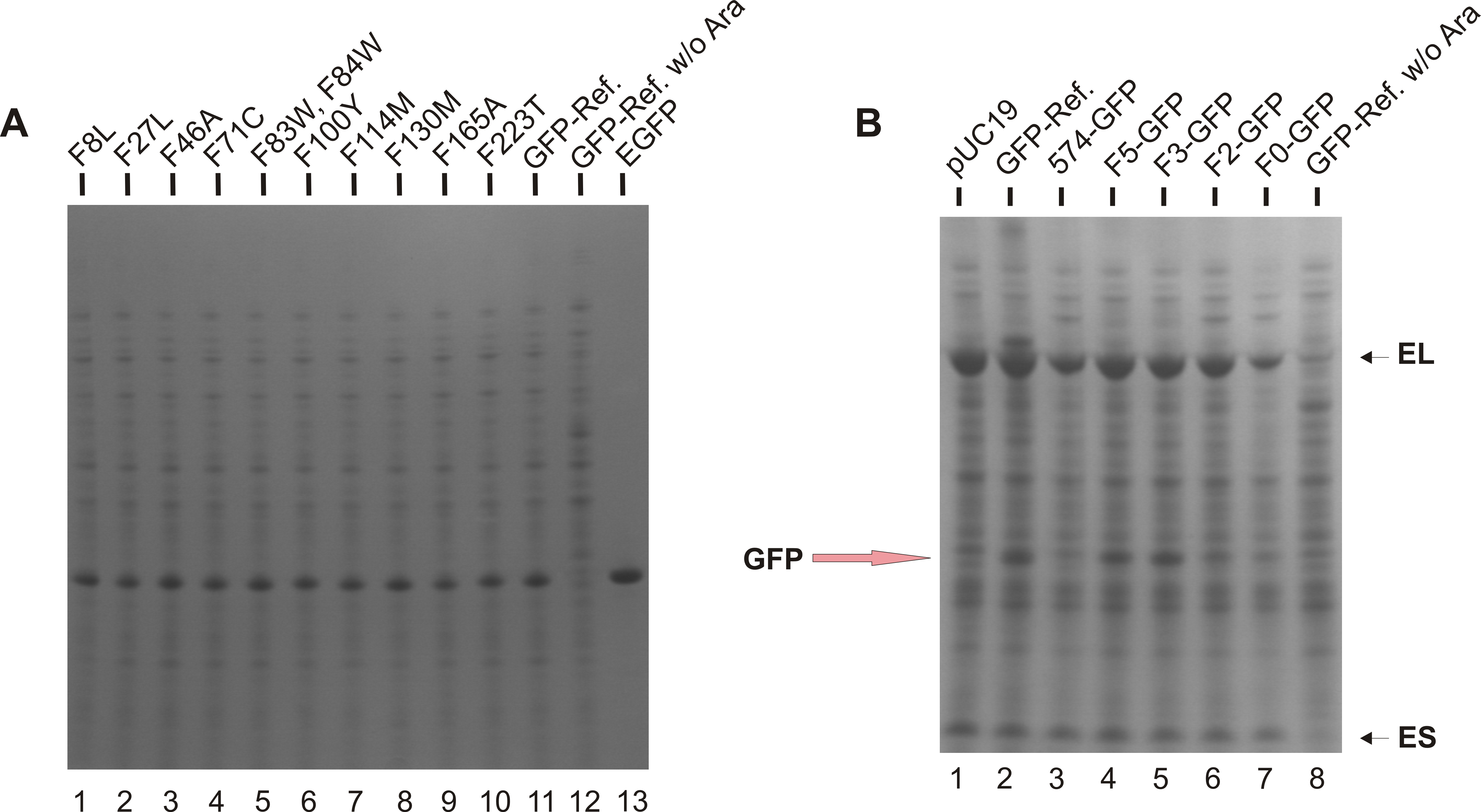

Supplement: Figure S3 — GFP abundance in whole cell lysates. Protein analysis by SDS-PAGE and coomasie staining of whole cell lysates from cultures expressing (A) single-substitution GFP mutants and (B) evolved GFP variants. EL and ES indicates GroEL and GroES, respectively. (3.01 MB TIF) [file pone.0010104.s007.tif]

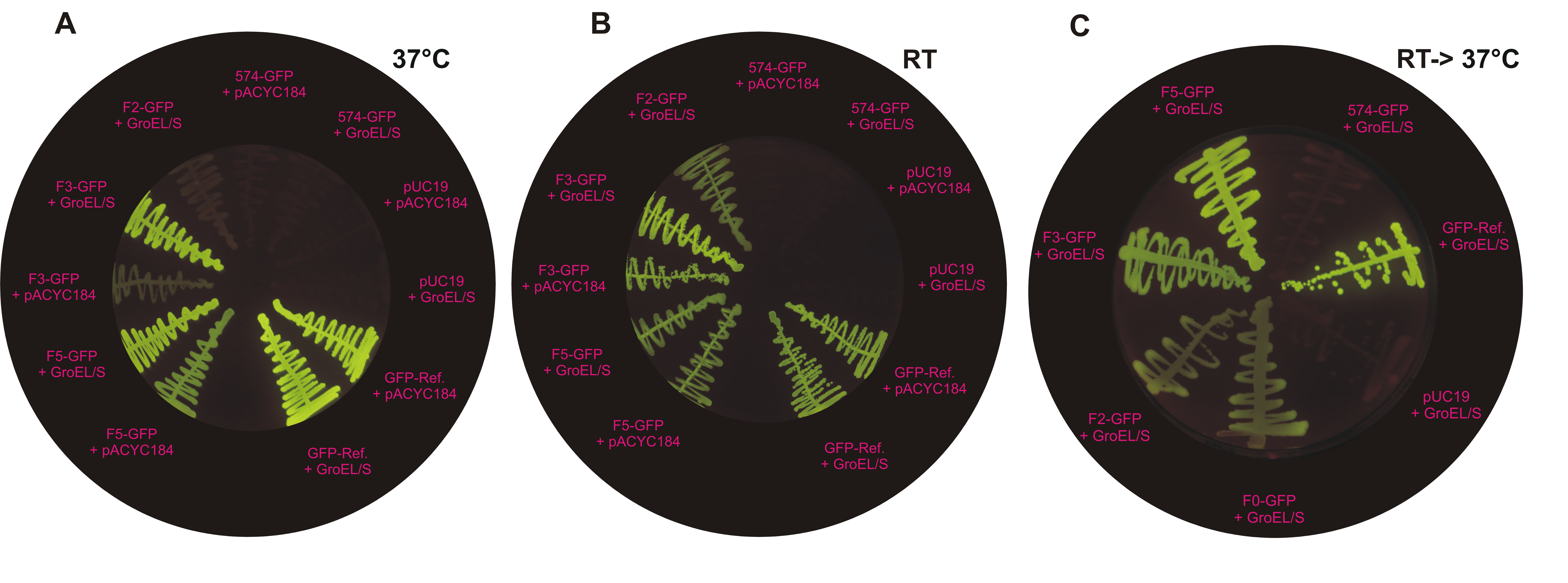

Supplement: Figure S4 — Chaperonin and temperature dependence of evolved GFP variants. DH5α expressing the indicated evolved GFPs and co-transformed with either pGro7 or pACYC184 were streaked on nitrocellulose placed on LB-agar plates containing ampicillin and chloramphenicol and grown overnight at 37° C. The filters were transferred to similar plates supplemented with 0.1% arabinose and incubated overnight at 37° C (A) or room temperature (B). (C) GFP mutants expressed at room temperature in the presence of GroES/L followed by transfer to 37° C and continued incubation for 24 h. pUC19 was used as a control as indicated. (5.64 MB TIF) [file pone.0010104.s008.tif]

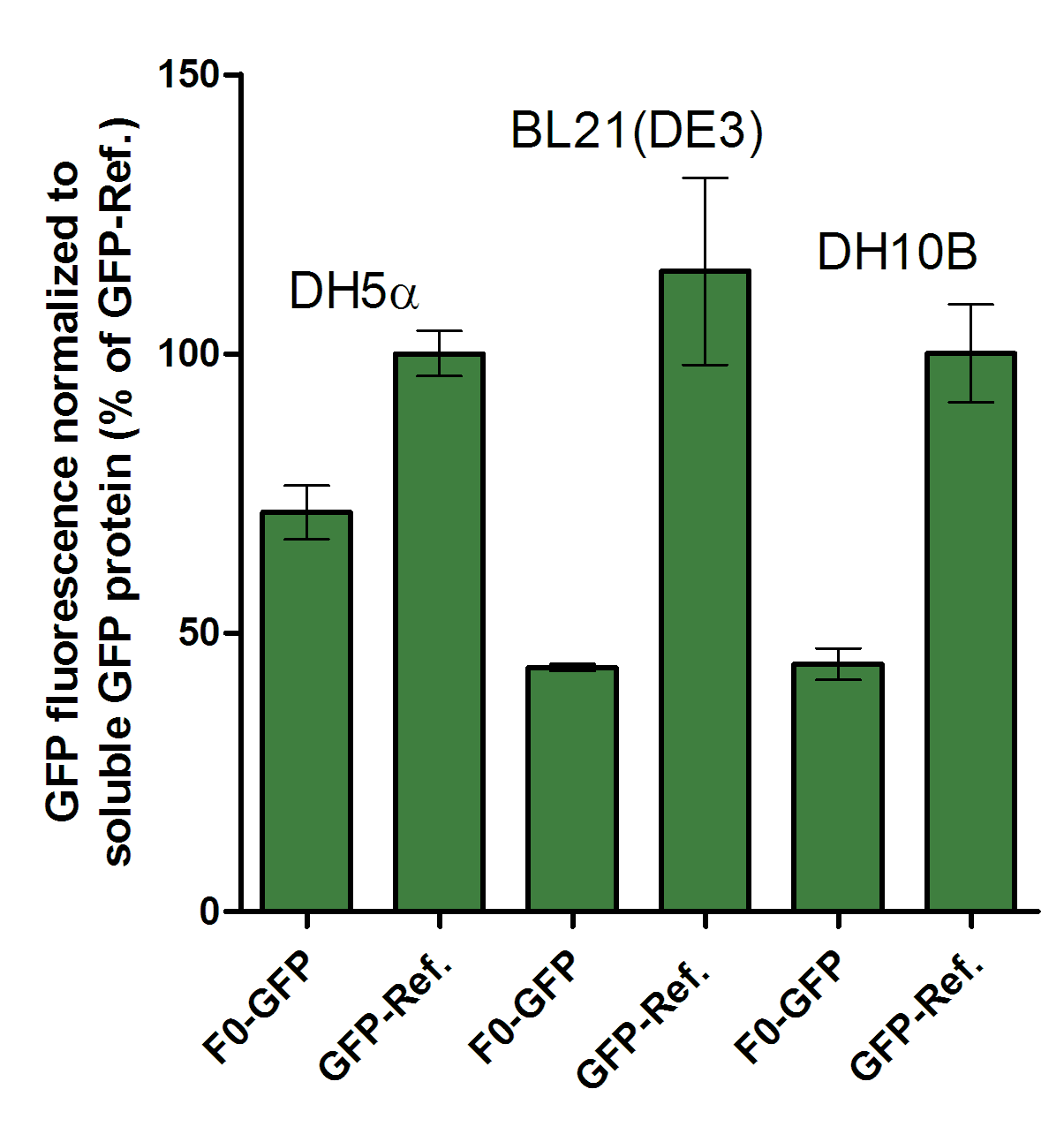

Supplement: Figure S5 — F0-GFP and GFP-Ref expression in three different strains. Fluorescence of overnight cultures co-expressing the indicated GFP variant and GroES/L in E.coli strains DH5α, BL21(DE3) and DH10B. Fluorescence and cell growth was monitored over time (18 h) at 23° C in the presence of 0.1% arabinose and the end level fluorescence was normalized against soluble GFP protein. Background fluorescence using a pUC19/DH5α culture was subtracted. The mean and SD of quadruplicate experiments is shown. (1.63 MB TIF) [file pone.0010104.s009.tif]

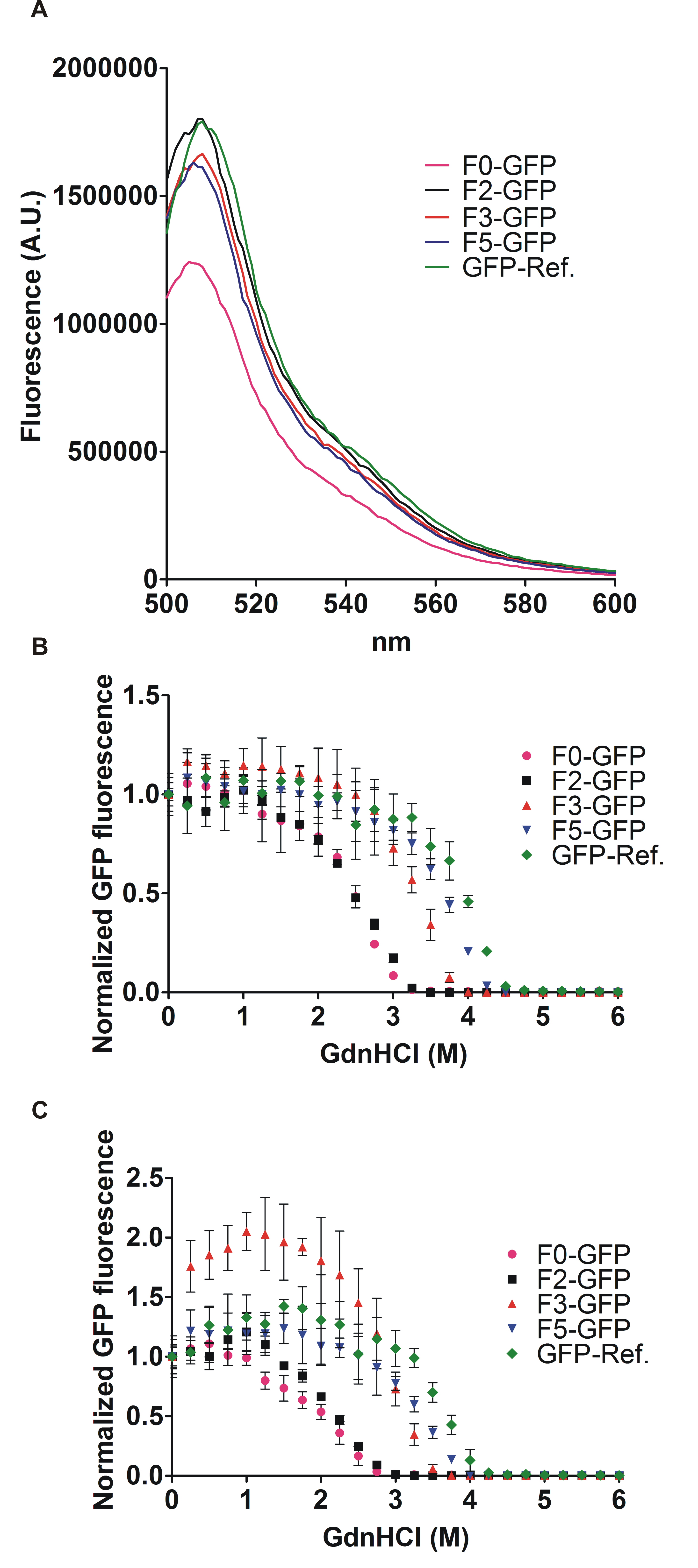

Supplement: Figure S6 — Biophysics of GFP Phe mutants. (A) Emission spectra of indicated GFP variants. (B and C) GdnHCl-unfolding titration at room temperature of the indicated GFP variants at 24 h (B) or 72 h (C) of incubation. The mean and SD of triplicate experiments is shown. (2.41 MB TIF) [file pone.0010104.s010.tif]
